# Intestinal Bacterial Diversity and Functional Analysis of Three Lepidopteran Corn Ear Worm Larvae

**DOI:** 10.3390/insects13080740

**Published:** 2022-08-17

**Authors:** Jiqiang Zhang, Shanshan Gao, Fangqiang Zheng, Ningxin Wang

**Affiliations:** College of Plant Protection, Shandong Agricultural University, Taian 271018, China

**Keywords:** *Conogethes punctiferalis*, intestinal bacteria, diversity, function, *Ostrinia furnacalis*, *Helicoverpa armigera*

## Abstract

**Simple Summary:**

Corn is one of the most important food crops in the world and comprises a large proportion of crops in China. Pests are one of the important factors affecting corn yield. *Conogethes punctiferalis*, *Ostrinia furnacalis* and *Helicoverpa armigera* are three main pests in the ear stages of corn, which significantly affect the yield and quality of corn. The three co-occurring lepidopteran pests at the ear stage occur frequently. Recently, the amount of *Conogethes punctiferalis* increased, even becoming the most serious pest in the Huang-Huai-Hai summer corn region of China, which is the second-largest corn producing area in China. Gut bacteria play important roles in insect adaptation to various environments. This study aimed to compare the diversity and function of the intestinal bacteria in three co-occurring lepidopteran pests, and to explore the reason for their prevalence. The results might provide insight into the prevalence of corn earworm larvae from the perspective of gut microbiota and function prediction.

**Abstract:**

Insects, as the most abundant animal group on earth, and their symbionts help their hosts to adapt to various environments. *Conogethes punctiferalis*, *Ostrinia furnacalis* and *Helicoverpa armigera* are three main pests co-occurring in the ear stage of corn, which significantly affect the yield and quality of corn. The purpose of this study was to compare the diversity and function of the intestinal bacteria of the three co-occurring lepidopteran pests, *C. punctiferalis*, *O. furnacalis* and *H. armigera*, and to explore the reason of their prevalence from the microbiota’s view. Our results showed the difference of diversity and abundance of the gut bacteria of three co-occurring lepidopteran pests at the ear stage. Proteobacteria and Firmicutes were the dominant phyla, and the Enterobacteriaceae and Enterococcaceae were the dominant families in the three pests. Compared with the other two pests, Bacteroidetes was found much more in *C. punctiferalis*. In addition, *C. punctiferalis* showed more correlation and similarity in bacteria composition with corn endophytic bacteria, as well as had obvious advantages in metabolic, environmental information processing, cellular processes and organic systems function pathways. Our findings may provide insight into the prevalence of corn earworm larvae from the perspective of gut microbiota and function prediction.

## 1. Introduction

Corn is the world’s most widely grown and highest-yielding food crop. There are six major corn areas in China. The Huang-Huai-Hai summer corn region is China’s second-largest corn-producing area, comprising about 40% of the country’s cultivated area and yields [1]. Despite many other factors influencing corn productivity, pests have always been an important component that affect corn production [2]. Due to increased corn planting area, industrialization, improved agricultural systems, global warming and other factors, corn pests have become more common in the 21st century, which has negatively impacted corn yields [3]. In China, there are more than 200 species of corn pests, with more than 10 of them capable of causing significant yield reductions, accounting for 10 to 20% of total corn production every year [4].

The ear stage of corn is from jointing to tasseling, which is a critical period for the growth and development of corn [3,5]. In recent years, the dominant pest population has shifted, and the degree of damage has increased. The infestations involving different pests are common, posing serious threats to corn growth, production and quality [6]. Therefore, the proper management at this stage is critical to corn yield. Lepidopteran pests are among the most significant and detrimental in the corn field, and they can directly impact corn productivity and quality [4]. *Conogethes punctiferalis* (Guenée), *Ostrinia furnacalis* (Guenée) and *Helicoverpa armigera* (Hübner) are three co-occurring lepidopteran pests in the ear stage [7,8]. *O. furnacalis* is commonly known as corn drill or stem borer and *O. furnacalis* was deemed as the most harmful pest of cultivated corn in China [9]. Corn is also an important host plant of *H. armigera* [10], which causes serious harm to the growth of summer corn. The yellow peach moth, *C. punctiferalis*, is a serious polyphagous pest of fruits (peach, plum, durian, etc.) and crops (corn, sorghum, cotton, etc.) [11]. Furthermore, the damage of *C. punctiferalis* has gradually increased recently, even more than *O. furnacalis* and *H. armigera*, especially in the Huang-Huai-Hai region [1,12]. All three pests are widely distributed in China [1,13,14].

Insects are the most numerous animal group on earth, and their symbionts play a crucial role in adapting to different environments. Microorganisms live in insect exoskeletons, intestines, other organelles and insect cells and have a close parasitic to a mutualistic relationship with hosts [15]. In the long-term process of coevolution, insects and their microorganisms have formed an interdependent symbiotic relationship. Insects provide a stable living environment and essential nutrients for microorganisms. In turn, intestinal bacteria are also involved in a variety of insect metabolic processes, providing insects with nutrients [16] and digesting complex carbohydrates [17], affecting the growth [18], development and reproduction of the host [16,19], and participating metabolism of the host [20,21]. Bacteria can also protect the host from pathogens, parasites or natural enemies by producing toxins or antibacterial compounds [22,23,24], regulating host adaptability [25,26,27]. Symbiotic bacteria also have important effects on drug resistance [28,29]. For example, bacterial symbiont from *Nilaparvata lugens* could affect insecticide (imidacloprid) resistance [29]. Overall, bacteria help insect hosts adapt to the changing environment in many ways.

Lepidoptera is the second largest insect order with some of the devastating agricultural pests worldwide [30], The community structure of intestinal microorganisms are affected by a variety of factors. Diet is an important factor. Different diets can change the abundance and diversity of intestinal microorganisms of *Grapholitha molesta* [17]. The composition of intestinal flora is also greatly influenced by host species. *Grapholita molesta*, *C. punctiferalis*, *Carposina sasakii* and *Cydia pomonella* eat the same food, yet there are big differences in the intestinal microflora [31]. Similarly, the environment also had a significant impact on the intestinal microbiota of lepidopteran pests, with significant differences in the intestinal microbiota between the field and laboratory populations of *H. armigera* [32].

In our current study, we used a high-throughput sequencing method to investigate the gut bacterial communities of three lepidoptera pests, *H. armigera*, *C. punctiferalis* and *O. furnacalis*, collected from the corn ear stage at the same time, as well as the healthy corn symbiont bacteria. We aim to find the bacterial relationship between the three co-occurring pests and get some information about the pests’ prevalence from a microbial perspective. The results may also provide some insights into the control and management of these pests.

## 2. Materials and Methods

### 2.1. Insects and Corn Samples

Three co-occurring lepidopteran pests of corn at ear stage, *H. armigera*, *C. punctiferalis* and *O. furnacalis* were collected from the corn experimental field of Tai’an, Shandong Province, China. Meanwhile, healthy corn grains were collected from the same location.

### 2.2. DNA Extraction

Twenty healthy 4th to 5th instar larva of 3 different corn earworms were selected for dissection as a sample, and each pest had five replicates. After 24 h of starvation, all individuals were surface-sterilized in 75% ethanol for 2 min, followed by three rinses in sterile water. Midgut dissections were conducted in a clean Petri dish (90 mm in diameter) by an anatomical microscope under a clean bench. Healthy and full corn seeds were selected, soaked in 75% alcohol for 2 min, rinsed with sterile water 3 times, then ground with sterile water and put 1 mL abrasive solution into a 1.5 mL centrifuge tube for later use.

Microbial community genomic DNA was extracted from samples using the OMEGA-D5625-01 Soil DNA Kit according to manufacturer’s instructions. The DNA extract was checked on with 1% agarose gel, and DNA concentration and purity were determined with NanoDrop 2000 UV-vis spectrophotometer (Thermo Scientific, Wilmington, NC, USA).

### 2.3. PCR Amplification and High-Throughput Sequencing

The hypervariable region V3-V4 of the bacterial 16S rRNA gene was amplified with primer pairs 338F (5-ACTCCTACGGGAGGCAGCAG-3) and 806R (5-GGACTACHVGGGTWTCTAAT-3) by an ABI GeneAmp^®^ 9700 PCR thermocycler (ABI, Los Angeles, CA, USA). The PCR amplification of the 16S rRNA gene was performed as follows: initial denaturation at 95 °C for 3 min, followed by 27 cycles of denaturing at 95 °C for 30 s, annealing at 55 °C for 30 s, extension at 72 °C for 45 s and single extension at 72 °C for 10 min, and end at 4 °C. The PCR mixtures contained 5 × TransStart FastPfu buffer 4 μL, 2.5 mM dNTPs 2 μL, forward primer (5 μM) 0.8 μL, reverse primer (5 μM) 0.8 μL, TransStart FastPfu DNA Polymerase 0.4 μL, template DNA 10 ng and finally ddH2O up to 20 μL. PCR reactions were performed in triplicate. The PCR product was extracted from 1% agarose gel and purified using the AxyPrep DNA Gel Extraction Kit (Axygen Biosciences, Union City, CA, USA) according to manufacturer’s instructions and quantified using Quantus™ Fluorometer (Promega, Madison, WI, USA). Build a sequencing library using NEXTFLEX Rapid DNA-Seq Kit. Qualified libraries were sequenced using Illumina Miseq PE300 by Majorbio Bio-Pharm Technology Co. Ltd. (Shanghai, China).

### 2.4. Statistical and Bioinformatics Analysis

In order to get high-quality readings, each sample were spliced through overlap by FLASH (Fast Length Adjustment of Short Read, version 1.2.11), a read pre-processing software, which assembled and merged the paired-end reads from fragments and generated >10 bp overlapped, with a maximum mismatch rate of the overlap region of 0.2 (https://ccb.jhu.edu/software/FLASH/index.shtml, (accessed on 12 August 2021)). Fastp (version 0.19.6, https://github.com/OpenGene/fastp, (accessed on 24 September 2021) was used for quality control of original sequencing to obtain optimized data. The non-repetitive sequences were extracted from the optimized sequences, which could reduce the computational complexity of the intermediate process. The single sequences without duplication were removed, and operational taxonomic units (OTUs) based on the non-repetitive sequences (excluding the single sequence) were clustered according to 97% similarity by using UPARSE (version 7.1, http://drive5.com/uparse/,(accessed on 27 September 2021). During the clustering process, chimeric sequences were removed, and the representative sequences of OTUs were obtained. After the above steps, the optimized sequence can be obtained for further analysis. All the optimized sequences were mapped to the OTU representative sequence, and the sequences with more than 97% similarity were selected to generate the OTU table. To accurately assess the diversity of the microbial communities, all samples were rarefied to the same depth based on the minimum sequence number. Sample data were homogenized using rarefaction by the “vegan” package in R (version 3.3.1). The subsequent analyses conducted in this study were based on normalized data. To obtain the information of the species corresponding to each OTU, the RDP classifier (version 2.11, https://sourceforge.net/projects/rdp-classifier/,(accessed on 17 October 2021) Bayesian algorithm was used to analyze 97% similarity of the OTU representative sequence against the Silva (version 138, https://www.arb-silva.de/, (accessed on 20 October 2021) ribosomal RNA gene database using a confidence threshold of 70%. The community composition of each sample was analyzed at each classification level. Majorbio’s cloud platform was used to extract the original data. The chloroplasts and mitochondria sequences were then removed by the platform and annotated the obtained sequences for species.

To count all OTUs and the number of OTUs shared and unique in multiple samples, Venn diagrams were made by the “venn diagram” package in R (version 3.3.1), and the sparse curves and other richness and diversity indices (ACE, Chao, Shannon and Simpson) of bacterial communities were estimated by Mothur (version 1.30.2, https://www.mothur.org/wiki/Download_mothur, (accessed on 15 November 2021). The Circos-0.67-7 (http://circos.ca/, (accessed on 21 December 2021) was used to make the Circos sample and species relationship map. Clustering was performed using the “vegan” package of R language according to the similarity of abundance among species or samples to make a community Heatmap diagram and a community Pie diagram (PIE diagram). Difference tests were performed on the multiple groups of samples, and the Kruskal–Wallis H test was used as well as the “stats” package of R (version 3.3.1) and the “scipy” package of Python for mapping. Qiime (version 1.9.1, http://qiime.org/install/index.html, (accessed on 19 January 2022) was used to calculate the Beta diversity distance matrix for Hierarchical clustering analysis. A UPGMA algorithm was used to construct the tree structure, using the “pheatmap” package in R (version 3.3.1) for plotting. Principal coordinate analysis (PCoA) based on the Bray-Curtis distance was applied to reveal the differences in bacterial communities between groups. LEfSe (http://huttenhower.sph.harvard.edu/galaxy/root?tool_id=lefse_upload, (accessed on 16 February 2022) was used for analysis and mapping, and Linear discriminant analysis (LDA) was used to screen the biomarkers for statistical differences between groups with LDA scores greater than 2. Use Networkx (version 2.1) network analysis kit to obtain the relative information of species and samples within or between groups and build a species correlation network. The Pathway (Level 1, Level 2, level 3) information in KEGG database was obtained by PICRUSt2 (version 2.2.0, https://github.com/picrust/picrust2/, (accessed on 26 March 2021) [33], and the function information of COG database and MetaCyc database was compared to predict the function of the assumed microbial community comprehensively. At the same time, a cluster heat map of the metabolic pathway abundance table was made by GraphPad Prism 8.0.2. Alpha analysis, Circos analysis, network analysis and function prediction were all calculated by each sample data, and the mean values of all samples of each insect species were then used for mapping and analysis. Raw sequencing data are available on the NCBI Sequence Read Archive under BioProject accession number PRJNA819209.

Differences were considered significant when * *p* < 0.05 and extremely significant when ** *p* < 0.01. SPSS23.0 software was used for statistical analysis.

## 3. Results

### 3.1. Analysis of 16S rDNA Sequencing Results

A total of 18 samples, including 15 insect gut samples and 3 corn samples, were sequenced by Illumina Miseq PE300 and obtained 1,423,375 pairs of reads (Table 1). After splicing, quality control and redundancy removal, clustering analysis (based on 97% sequence similarity), and chimerism resulted in the removal of the above data. To accurately assess the diversity of the microbial communities, all samples were rarefied to the same depth based on the minimum sequence number. Sequence numbers were normalized to 12,358 for all samples of bacteria. Cluster analysis (based on 97% sequence similarity) obtained 176 OTUs, including 7 phyla, 16 classes, 31 orders, 67 families, 113 genera and 140 species. The Shannon–Wiener curves eventually flattened, indicating that the sequencing depth was sufficient to meet the requirements of the subsequent data analysis (Figure S1). Among the 176 OTUs, 106 were found in the intestinal of *C. punctiferalis*, 101 OTUs in the intestinal of *O. furnacalis* and 75 OTUs in the intestinal of *H. armigera* (Table S1), whereas 72 OTUs were the endophytic corn bacteria (Table S2). *C. punctiferalis* showed more OTU numbers than the other two insects. Compared with *H. armigera*, *C. punctiferalis* and *O. furnacalis* were closer in OTU numbers.

In the Venn diagram of the three lepidopteran pests, 42 OTUs were shared, whereas 29, 23 and 18 OTUs were specific to *C. punctiferalis*, *O. furnacalis* and *H. armigera*, respectively (Figure S2). Of note, *C. punctiferalis* exhibited the most specific OTUs. *C. punctiferalis* and the *O. furnacalis* shared more OTU numbers than the comparisons between the others.

### 3.2. Comparison of the Gut Microbiota

**Figure 1:**
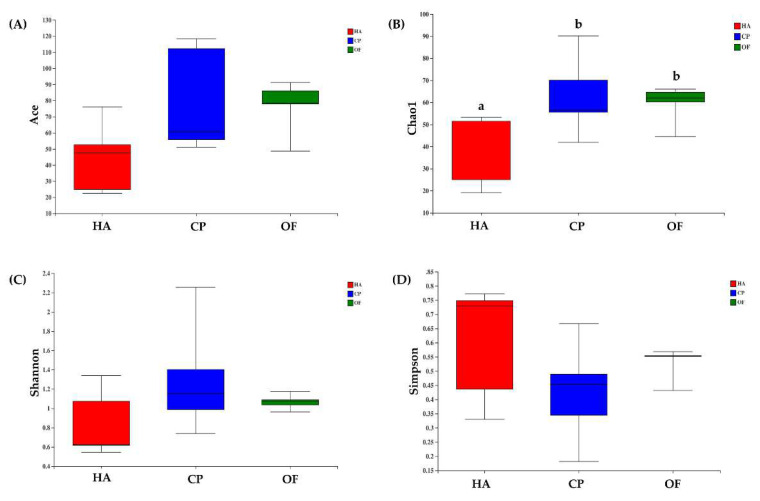
Box plots of (**A**) ACE, (**B**) Chao, (**C**) Shannon and (**D**) Simpson values of bacterial diversity in larval gut samples. Abbreviations in the figure are given following the scientific names of insects: HA, *H. armigera*; CP, *C. punctiferalis*; OF, *O. furnacalis*. Letters above each group indicate significant differences (*p* < 0.05) in the mean values.

The alpha diversity index indicated differences in intestinal flora of the three lepidopteran insects. The ace and chao indexes showed that the value of *C. punctiferalis* was the highest, followed by *O. furnacalis*, and *H. armigera* was the lowest. The values of *C. punctiferalis* and *O. furnacalis* in the Chao index were significantly different from those of *H. armigera* (Figure 1A,B). The Shannon index showed that *C. punctiferalis* had the highest value, followed by *O. furnacalis*, whereas *H. armigera* had the lowest value (Figure 1C). The Simpson index showed that *C. punctiferalis* had the lowest value, followed by *O. furnacalis*, and *H. armigera* had the highest value (Figure 1D). All the indexes above showed that the richness and diversity of the microbial community in *C. punctiferalis* were the highest.

Less than 0.1% abundance of the species presented at all levels (phylum, class, order, family, genus, species) and were grouped into “other”, and we calculated the community composition from different levels. Firmicutes and Proteobacteria (98.61 ± 0.59%) were the dominant phyla in the three pests (Figure S3). However, Proteobacteria and Firmicute showed differences among the three pests. Firmicute accounted for the majority (76.35%) and Proteobacteria less (22.61%) in *O. furnacalis* (Figure S5C). In *C. punctiferalis*, Firmicute accounted for 52.27%, and Proteobacteria accounted for 45.75% (Figure S5B). Additionally, in *H. armigera*, Proteobacteria and Firmicute accounted for 62.53% and 36.99% (Figure S5A), respectively. Of note, Bacteroidetes was significantly different, at less than 0.1% in *O. furnacalis* and *H. armigera*, whereas it was 1.6% in *C. punctiferalis* (Figure S5).

At the family level, Enterobacteriaceae and Enterococcaceae (83.68%, 98.46% and 84.90% in *O. furnacalis*, *H. armigera* and *C. punctiferalis*, respectively) were the main dominant families (Figure S6). Enterococcaceae (70.50%) was the absolute dominant family in *O. furnacalis*, followed by Enterobacteriaceae (13.18%) (Figure S6C). In *H. armigera*, Enterobacteriaceae (61.91%) was the most dominant family, and Enterococcaceae (36.55%) was the second, whereas the other family was less than 1% (Figure S6A). Enterococcaceae (52.01%) and Enterobacteriaceae (32.89%) were the top two in *C. punctiferalis* (Figure S6B). Moraxellaceae, Sphingobacterium, Pseudomonadaceae, Flavobacteriaceae, Pseudomonas and Alcaligenaceae were much more abundant in the *C. punctiferalis* intestinal tract than the other two pests, and Alcaligenaceae was almost exclusively present in the gut of *C. punctiferalis* (Figure S4). Among them, Alcaligenaceae (*p* = 0.04138) showed significant differences in comparing the three samples (Figure S9A).

**Figure 2:**
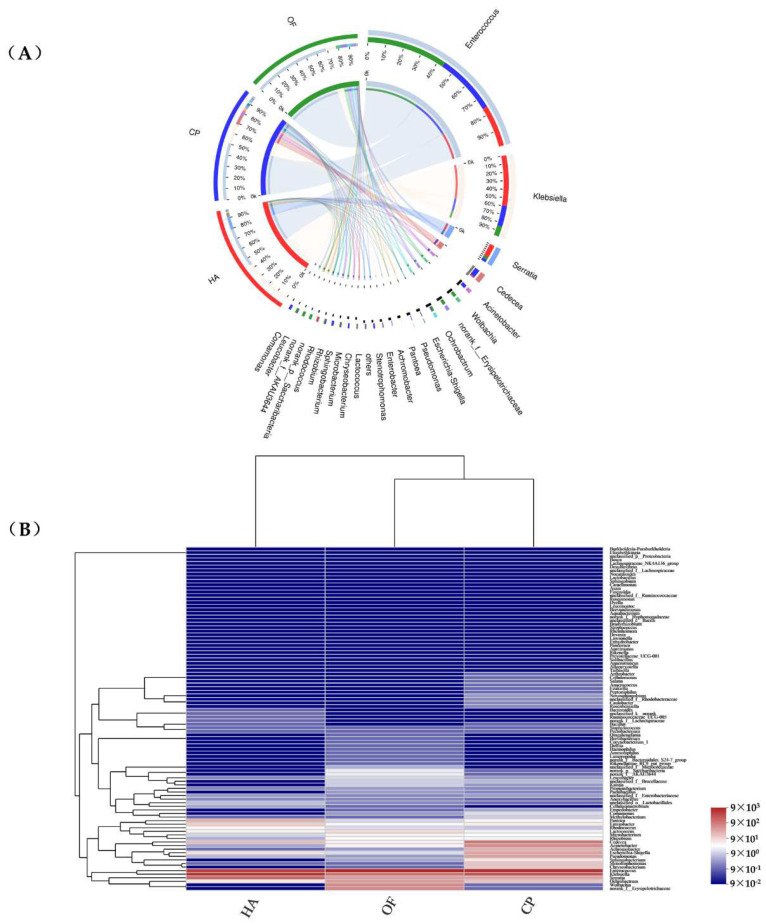
Circos (**A**) and distance heatmap (**B**) based on genus level. Circos (**A**) shows distribution proportion of dominant species in each sample and distribution proportion of dominant species in different samples. The columns (**B**) represent the samples and the rows represent the bacterial assigned to the genus level. Dendrograms of hierarchical cluster analysis grouping genera and samples are shown on the left and bottom, respectively. The color scale represents the normalized values of relative abundances by log10. HA, *H. armigera*; CP, *C. punctiferalis*; OF, *O. furnacalis*. Taxa with an abundance <1% are included in ‘Other’.

Enterococcus and Klebsiella (79.78%, 81.11% and 70.40% in *O. furnacalis*, *H. armigera* and *C. punctiferalis,* respectively) were the dominant bacterial genera in the three lepidopterans pests (Figure 2A), showing a high abundance in the heatmap. Enterococcus (70.50%), Klebsiella (44.56%) and Enterococcus (52.01%) were the most dominant genera in *O. furnacalis*, *H. armigera* and *C. punctiferalis*, respectively (Figure S7). The abundance of Cedecea, Acinetobacter, Achromobacter, Escherichia-Shigella, Pseudomonas, Sphingobacterium, Stenotrophomonas and Chryseobacterium in that peach moth was higher than that of the other two pests (Figure 2B). Achromobacter (*p* = 0.04138) showed significant differences among the three lepidopteran pests (Figure S9B).

OTU105 (Enterococcus) and OTU151 (Klebsiella) were found in three pests with a large proportion and high abundance. These two OTUs together occupied 70.36%, 79.63% and 80.81% of *C. punctiferalis*, *O. furnacalis* and *H. armigera*, respectively. The abundance of OTU2 (Enterobacter) (8.69%) in *C. punctiferalis* was higher than that of the *O. furnacalis* and *H. armigera* (Figure S8). The OTU49 (Serratia) (3.02%, 12.57% and 2.62% in *O. furnacalis*, *H. armigera* and *C. punctiferalis*) was also highly abundant in the three pests. OTU42 (Acinetobacter) (*p* = 0.0439), OTU103 (Pseudomonas) (*p* = 0.04863) and OTU1 (Achromobacter) (*p* = 0.04138) were abundant in the *C. punctiferalis*, and they showed significant differences in the overall comparison of the three lepidopteran pests (Figure S9C).

**Figure 3:**
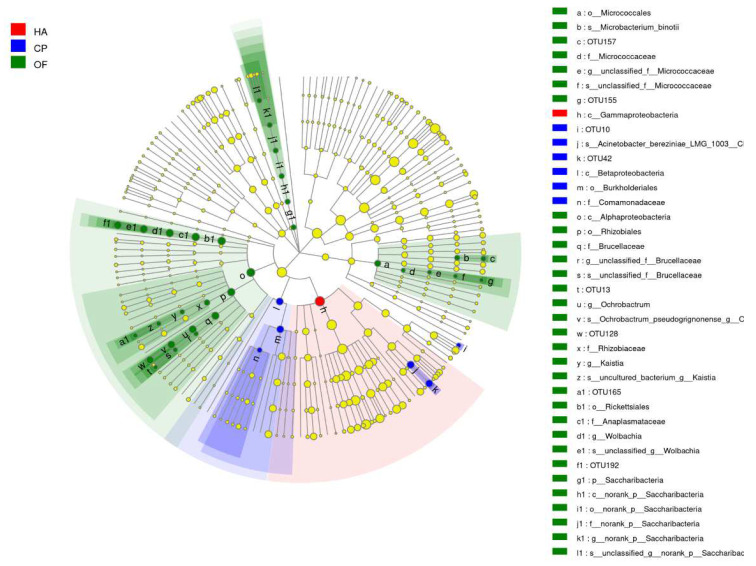
Cladogram of bacterial biomarkers, from the phylum (innermost ring) to species (outermost ring) level, with an LDA score >2. Each small circle at different taxonomic levels represents a taxon at that level, and the diameter of the circle is proportional to the relative abundance. The coloring principle is to color the species with no significant difference as yellow and the other different species as the group with the highest abundance of the species. Different colors represent different groups, and nodes with different colors represent the communities that play an important role in the group represented by the color. HA, *H. armigera*; CP, *C. punctiferalis*; OF, *O. furnacalis*.

Cladogram from phylum to species was drawn to fully understand the distribution of these different taxa at various taxonomic levels (Figure 3). It was found that the community differences of the three lepidopteran pests were mainly concentrated in Proteobacteria, Actinobacteria and Saccharibacteria, and Proteobacteria caused the most significant difference. One taxon differed significantly in the gut microbiota of *H. armigera*, whereas 4 and 25 taxa differed significantly in *C. punctiferalis* and *O. furnacalis* (Figure S10).

### 3.3. Beta Diversity Analysis

At the genus level, the three pests were clustered together from the hierarchical clustering tree based on the Bray-Curtis distance algorithm, although the clustering form was chaotic. However, the corn samples were clustered in the same branch, which means that the composition similarity of the flora was higher than that of the three pests. The *C. punctiferalis* samples were the most complex, with some samples clustered with *H. armigera* or *O. furnacalis* and some samples clustered on one branch alone (Figure S11).

**Figure 4:**
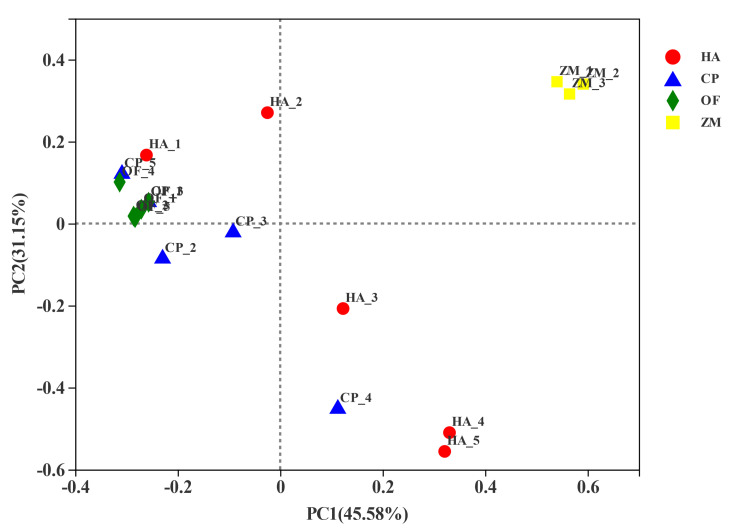
PCoA analysis based on Bray-Curtis distance algorithm. HA, *H. armigera*; CP, *C. punctiferalis*; OF, *O. furnacalis*.

The PCoA analysis based on the Bray-Curtis distance algorithm was used to compare the community similarities between samples. The PCoA scatter plot showed that the abscissa and ordinate represent the two characteristic values contributing to the largest differences between the samples. Their influence degrees were 45.58% and 31.15%, respectively (Figure 4). The corn endophytic bacteria flora composition at the genus level differed from that of the pests. The corn endophytic bacteria flora was gathered together, whereas the bacterial flora of three pests covered more (Figure 4).

Performing subsequent correlation network analysis based on the selected samples with the sample abundance greater than 10, the collinear network analysis was carried out on three pests and corn. In addition to the common bacteria shared by the corn and three pests, the *C. punctiferalis* and corn samples were associated with Bacteroidetes at the phylum level (Figure S12A). They were also associated with Pseudomonadaceae, Flavobacteriaceae and Xanthomonadaceae at the family level (Figure S12B). At the genus level, they were associated with Chryseobacterium, Pseudomonas (Figure S12C), and they shared OTU103 at the OTU level (Figure S12D).

### 3.4. Functional Prediction of Gut Microbiota

**Figure 5:**
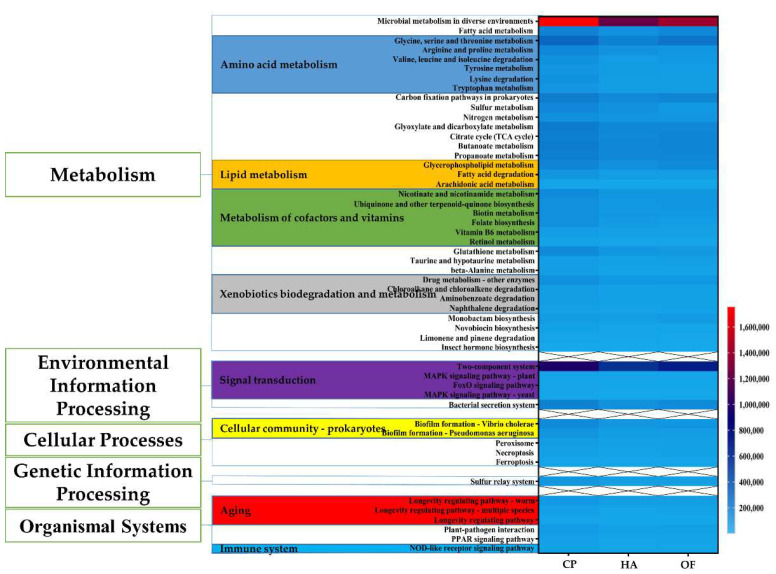
Comparison of KEGG pathway function prediction of intestinal microbial community. The left side is the pathway Level 1 function, and the middle is the pathway Level 2 function, while the right heatmap is the pathway Level 3 function. The pathway Level 2 function marked with various colors are all functions with significant differences, and the second-class function marked with different colors is also a function with significant differences. The scale on the right corner represents the abundance of the functions in each group. HA, *H. armigera*; CP, *C. punctiferalis*; OF, *O. furnacalis*.

To understand the function of gut microbes of the three panicle pests, a functional assessment through PICRUSt2 was performed to analyze the KEGG pathway. Compared with the other two pests, *C. punctiferalis* showed higher abundance at three different levels. At Level 1, the abundance of metabolism pathways was higher than other pathways, such as environmental information processing, cellular processes, organismal systems, etc. Metabolism plays an important part in organisms. At Level 2 under metabolism Level 1, amino acid metabolism, metabolism of cofactors and vitamins, lipid metabolism and xenobiotics biodegradation and metabolism and metabolism of other amino acids all were superior in numbers in *C. punctiferalis* to that in *H. armigera* and *O. furnacalis* by the Homogeneity of variance test. Using the Homogeneity of variance test, similarly, at Level 3 under xenobiotics biodegradation and metabolism Level 2, drug metabolism-other enzymes, chloroalkane and chloroalkene degradation, aminobenzoate degradation and naphthalene degradation all showed predominance and significant difference in *C. punctiferalis* than the other pests (Figure 5). From biofilm formation-vibrio cholerae, biofilm formation-pseudomonas aeruginosa, under Level 2 of cellular community–prokaryotes, and Level 2 of environmental adaptation, *C. punctiferalis* showed an obvious advantage over the other pests. In addition, *C. punctiferalis* showed significant differences in microbial metabolism in diverse environments (Level 3), fatty acid metabolism (Level 3), sulfur metabolism (Level 3), bacterial secretion system (Level 3), necroptosis (Level 3) and insect hormone biosynthesis (Level 3). However, they were not significant at corresponding Level 2 (Figure 5).

Furthermore, *C. punctiferalis* revealed a high abundance in carbohydrate metabolism (Level 2), membrane transport (Level 2), biosynthesis of other secondary metabolites (Level 2), glycan biosynthesis and metabolism (Level 2), transport and catabolism (Level 2), metabolism of terpenoids and polyketides (Leve l2), biosynthesis of other secondary metabolites (Level 2), energy metabolism (Level 2), digestive system (Level 2) and the excretory system (Level 2). Compared with *H. armigera*, the abundance of some functions in *O. furnacalis* was closer to the *C. punctiferalis*. It even exceeded the *C. punctiferalis* at translation (Level 2), transcription (Level 2) and cellular Community-eukaryotes (Level 2) (Figure S13).

## 4. Discussion

In the corn ear stage, *C. punctiferalis*, *H. armigera* and *O. furnacalis* are three co-occurring pests that seriously affect the corn yield [34,35,36]. Recently, the *C. punctiferalis* has gradually aggravated and surpassed the *O. furnacalis* and *H. armigera* as the most important pest, especially in the Huang-Huai-Hai summer corn region [1]. In this study, we investigated the bacterial diversity and community composition of the three pests and the corn endophytic bacteria. According to our findings, *C. punctiferalis* gut bacteria composition and activity and its close relationship with corn endophytic bacteria may explain its prevalence.

Previous studies have shown that Proteobacteria and Firmicutes are the dominant phyla in intestinal samples of many insects [37,38]. They were also reported to be dominant in lepidopteran insects [30], as we reported in this study. The most significant difference among these three lepidopteran pests was Bacteroidetes, which were much more abundant in the peach moth than in the other two insects. The dominant family of these three lepidopteran pests was Enterobacteriaceae and Enterococcaceae, whereas *Enterococcus* and *Klebsiella* were the main genera, which are consistent with the intestinal flora of *H. armigera*, *C. punctiferalis* and *O. furnacalis* studied before [31,32,39].

Previous studies showed that diet [16] and taxonomy could influence insect gut bacterial communities [38,40]. Although there were some differences between the three pests at various bacterial levels, the majority were not significant, most likely because of their similar diets and feeding times. Two Pyralidae insects, *C. punctiferalis* and *O. furnacalis* [41], showed a more similar number and abundance of bacteria families, genera and OTUs than the Noctuidae insect *H. armigera* [42], which means that the phylogeny plays an important role in shaping insect gut microbiota.

Correlation network analysis showed more relationships between *C. punctiferalis* and corn kernel. Because they shared more OTU, genus and family numbers from Proteobacteria and Bacteroidetes, the *C. punctiferalis* intestinal microflora had more correlation and similarity in bacteria composition with corn samples, which could reflect the *C. punctiferalis* intestinal microflora’s better adaptability to corn.

Among the three lepidopteran pests, OTU105 (*Enterococcus casseliflavus*), OTU151 (*Klebsiella pneumoniae*) and OTU49 (*Serratia marcescens*) were found in large amounts and accounted for a large proportion of the three lepidopteran pests. OTU105 (*E. casseliflavus*) was reported to have a complete L-tryptophan pathway in the silkworm intestine and could produce L-tryptophan, which is an essential aromatic amino acid for animal growth and development [43]. OTU151 (*K. pneumoniae*) may play important roles in fitness. It was reported *K. pneumoniae* could produce active molecules with effective antibacterial properties in cockroach intestines [44]. OTU49 (*S. marcescens*) is usually harmful and has strong pathogenicity and virulence [45,46]. *Serratia* was a pathogen in *H. armigera* [47]. In our study, *C. punctiferalis* showed the least *Serratia*, which is estimated to be related to the inhibition and antagonism of intestinal microorganisms.

*Conogethes punctiferalis* had a substantially higher abundance in functional prediction than the other two pests. On Level 1, the metabolism, environmental information processing, cellular processes and organismal systems were most outstanding in *C. punctiferalis*. Firstly, some intestinal bacteria of *C. punctiferalis* have been proved to have the ability to decompose and degrade the lignin, cellulose and hemicellulose [48,49]. In addition, they can use a variety of sugars, which is important for insects to overcome the defense mechanism of plants and adapt to complex environments and survive well. Proteobacteria exist widely in Lepidopteran insects and play important roles in function. *Cedecea lapagei* (OTU2), *Pseudomonas mosselii* (OTU103), *Pseudomonas hibiscicola* (OTU24) and *Acinetobacter pittii* (OTU38) all belong to Proteobacteria. OTU38 belongs to the *Acinetobacter* genus, which was reported to be able to degrade cellulose [50] and showed high total cellulase hydrolysis activity. Moreover, *A. pittii* was also found to have crude xylanase and magnetic xylanase (CLEA), which could convert xylanase form powdery rice straw (over 45%) and corn cob (over 60%) to xylo-oligosaccharide [49]. Secondly, some intestinal bacteria of *C. punctiferalis* could make good use of sugars. For example, *P. mosselii* could use a variety of sugars [51]. The *A. pittii* showed high endoglucanase hydrolytic activity [49]. All of the above functions enable insects to decompose better and digest food more easily, which perfectly corresponded with our predicted function higher in *C. punctiferalis*, glycan biosynthesis and metabolism (Level 2), transport and catabolism (Level 2) and the digestive system and excretory system (Level 2). Thirdly, the intestinal bacteria of *C. punctiferalis* have also shown adaptation and drug resistance. *Chruseobacterium cucumeris* (OTU200) belongs to Bacteroides. *C. cucumeris* and is involved in the biosynthesis of several compounds. It also contains genes for sodium/proton antiporter, glutathione, superoxide dismutase and cold shock proteins, which help it survive osmotic, oxidative and cold shock stress [52]. Moreover, *P. mosselii*, *P. hibiscicola*, *A. pittii* and *C. sediminis* could also degrade heavy metals, polycyclic aromatic hydrocarbons, long-chain alkanes and polychlorinated biphenyls [53,54,55,56,57,58]. It is speculated that they can help the host adapt to more complex environments and resist harsh ones. *C. lapagei* (OTU2), *Achromobacter ruhlandii* (OTU1) and *P. hibiscicola* (OTU24) were also reported to have resistant genes [59,60,61,62]. Similarly, the higher environmental adaptability and resistance of intestinal bacteria from *C. punctiferalis* were confirmed by functional analysis, such as xenobiotics biodegradation and metabolism (Level 2), drug metabolism-other enzymes (Level 3), chloroalkane and chloroalkene degradation (Level 3), aminobenzoate degradation (Level 3), naphthalene degradation (Level 3), environmental adaptation (Level 2), metabolism of terpenoids and polyketides (Level 2).

Furthermore, the intestinal bacteria of *C. punctiferalis* showed obvious bacteriostasis. *C. cucumeris* (OTU200) showed broad-spectrum antimicrobial activity [60]. *P. mosselii* (OTU103) and *P. hibiscicola* (OTU24) were reported to have an antagonistic effect on a variety of bacteria, including some pathogenic bacteria [63,64], which was speculated to better protect the *C. punctiferalis* from the invasion of some pathogenic microorganisms. Less *Serratia* observed in the stomach of *C. punctiferalis* compared to the other two pests may be explained by the presence of the above bacteria, implying that *C. punctiferalis* can readily withstand several pathogenic bacteria from the external environment, which is consistent with the functional prediction. *C. punctiferalis* was predicted with more xenobiotics biodegradation and metabolism (Level 2) and biosynthesis of other secondary metabolites (Level 2) functions.

In addition, *C. punctiferalis* gut bacteria also showed powerful metabolic ability. *P. mosselii* (OTU103) can produce bioactive secondary metabolites [65] and change the epithelial permeability of different cells to destroy the sytoskeleton [66]. *A. pittii* (OTU38) is also involved in unsaturated fatty acid synthesis, osmosis, membrane protein and expression of some genes in sulfur metabolism [56]. *C. punctiferalis* was predicted with more amino acid metabolism (Level 2), metabolism of other amino acids (Level 2), carbohydrate metabolism (Level 2), membrane transport (Level 2), lipid metabolism (Level 2), glycan biosynthesis and metabolism (Level 2), biosynthesis of other secondary metabolites (Level 2), metabolism of terpenoids and polyketides (Level 2) and transport and catabolism (Level 2) functions.

The intestinal bacterial composition and predicted functions of the three corn pests at the ear stage may explain the prevalence of *C. punctiferalis*. The increase amount of *C. punctiferalis* might be related to the abundant metabolic functions of *P. mosselii* (OTU103), *C. lapage* (OTU2), *A. ruhlandii* (OTU1), *P. hibiscicola* (OTU24) and other gut bacteria. However, metagenomic and meta-transcriptomic analysis will be needed in the future to elucidate host-microorganism interactions.

## 5. Conclusions

Our results showed differences in the gut bacteria communities of three co-occurring lepidopteran pests at the ear stage. Proteobacteria and Firmicutes were the dominant phyla, whereas the Enterobacteriaceae and Enterococcaceae were the dominant family in *C. punctiferalis*, *H. armigera* and *O. furnacalis*. Bacteroidetes were substantially more abundant in *C. punctiferalis* than in the other two insects, indicating a significant difference. *C. punctiferalis* had more correlation and similarity in bacteria composition with corn samples. According to the function prediction, the metabolism, environmental information processing, cellular activities, and organic systems dominated *C. punctiferalis*.

## Figures and Tables

**Table 1:** Summary of sequence statistics for the Illumina MiSeq runs for all samples.

Sample\Info	Seq_Num	Base_Num	Mean_Length	Min_Length	Max_Length
HA_1	94,956	42,807,665	451	280	452
HA_2	97,686	43,966,345	450	274	502
HA_3	102,225	45,948,879	449	388	455
HA_4	44,224	19,901,174	450	424	499
HA_5	42,012	18,903,405	450	362	454
CP_1	90,727	40,862,237	450	271	452
CP_2	103,449	46,502,122	450	266	471
CP_3	95,403	42,844,770	449	243	455
CP_4	31,459	14,157,568	450	423	454
CP_5	41,857	18,862,255	451	271	487
OF_1	100,844	45,255,687	449	274	476
OF_2	96,632	43,290,905	448	274	464
OF_3	96,775	43,465,463	449	274	499
OF_4	96,328	42,869,068	445	308	466
OF_5	102,951	46,293,847	450	351	489
ZM_1	110,385	47,802,629	433	379	452
ZM_2	33,976	14,943,705	440	401	494
ZM_3	41,486	17,993,290	434	403	501
SUM/AVG	1,423,375	636,671,014	447	326	473

The above data were all quality controlled and spliced. Sample\Info is the Sample name, Seq_num is the sequence number, Base_num is the base number and Mean_length is the average sequence length of all samples. Min_length is the shortest sequence length in the sample, and Max_length is the longest sequence length in the sample.

## Data Availability

The data presented in this study are available in supplementary material here.

## Supplementary Materials

The following supporting information can be downloaded at: https://www.mdpi.com/article/10.3390/insects13080740/s1, Figure S1: Shannon rarefaction curves for all samples; Figure S2: Venn diagram at OTU level; Figure S3: Circos (A) and distance heatmap (B) based on phylum level; Figure S4: Circos (A) and distance heatmap (B) based on family level; Figure S5: Pie chart of the intestinal and corn microbial community at phylum level; Figure S6: Pie chart of the intestinal and corn microbial community at family level; Figure S7: Pie chart of the intestinal and corn microbial community at genus level; Figure S8: Pie chart of the intestinal and corn microbial community at OTU level; Figure S9: Significance difference test based on Kruskal-Wallis rank sum test method at family level (A), genus level (B) and OTU level (C); Figure S10: Linear discriminant analysis (LDA) of three pests; Figure S11: Clustering analysis based on Bray-Curtis distance algorithm at genus level; Figure S12: Co-occurrence network diagram of all samples at phylum level (A), family level (B), genus level (C) and OTU level (D); Figure S13: Pathway heatmap of Level2; Table S1: Summary of multistage annotation information for three insect samples; Table S2: Summary of multi-level annotation information of corn samples.
