# Supplementary material for: Intestinal Bacterial Diversity and Functional Analysis of Three Lepidopteran Corn Ear Worm Larvae
Source: Insects. 2022 Aug 17;13(8):740. doi: 10.3390/insects13080740 (PMC9409944; doi:10.3390/insects13080740)
Supplement: Supplementary file 1 [file insects-13-00740-s001.zip › insects-1833986-supplementary.pdf]

Supplementary Figures

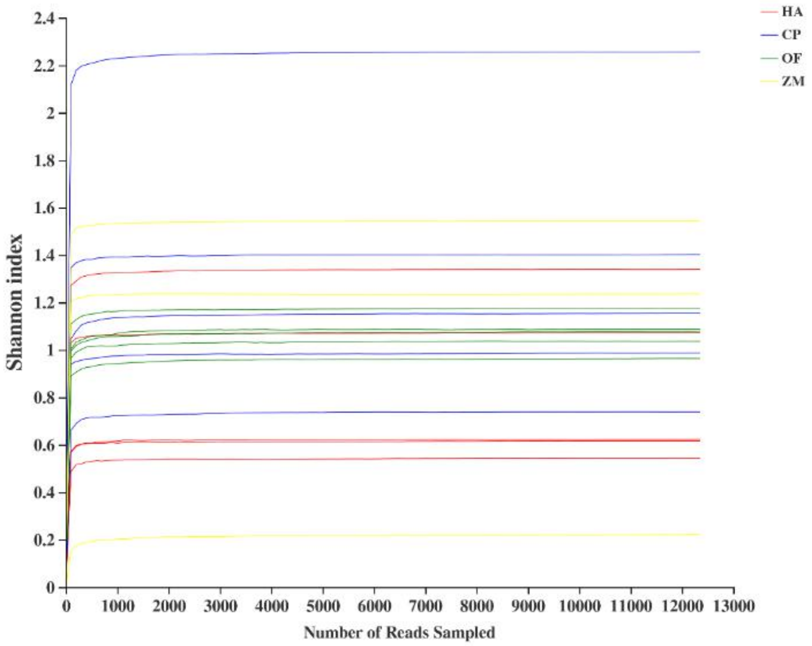

Figure S1. Shannon rarefaction curves for all samples. HA, *H. armigera*; CP, *C. punctiferalis*; OF, *O. furnacalis;* ZM, *Z. mays*.

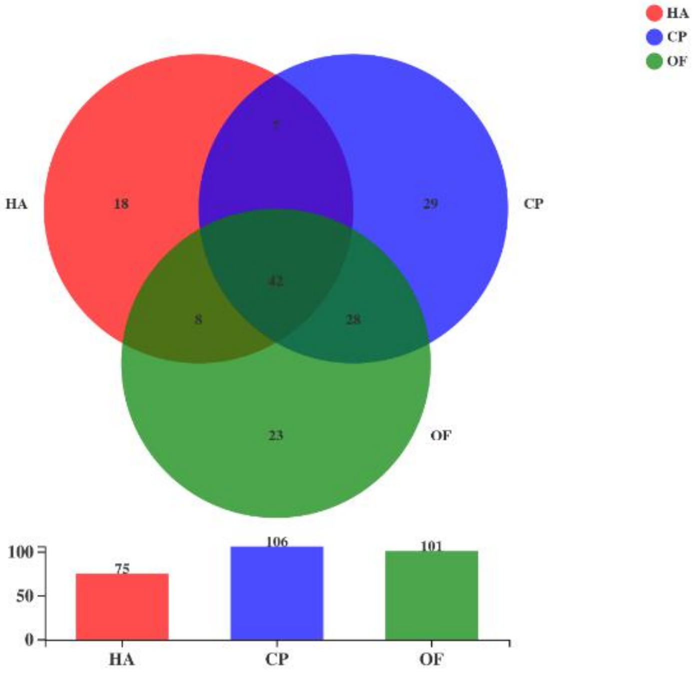

Figure S2. Venn diagram at OTU level. HA, *H. armigera*; CP, *C. punctiferalis*; OF, *O. furnacalis*.

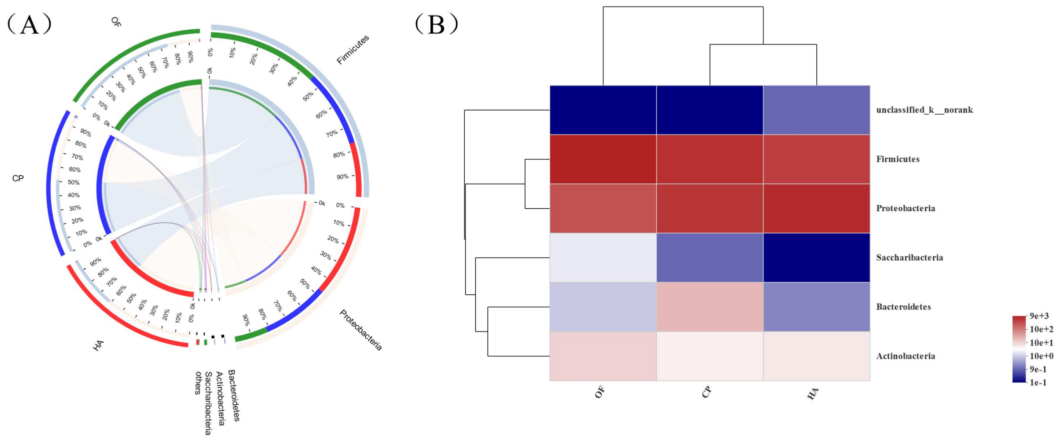

Figure S3. Circos (A) and distance heatmap (B) based on phylum level. Circos (A) shows distribution proportion of dominant species in each sample and distribution proportion of dominant species in different samples. The columns (B) represent the samples and the rows represent the bacterial assigned to the phylum level. Dendrograms of hierarchical cluster analysis grouping genera and samples are shown on the left and at the bottom, respectively. The color scale represents the normalized values of relative abundances by log10. HA, *H. armigera*; CP, *C. punctiferalis*; OF, *O. furnacalis*. Taxa with an abundance < 1% are included in 'Other'.

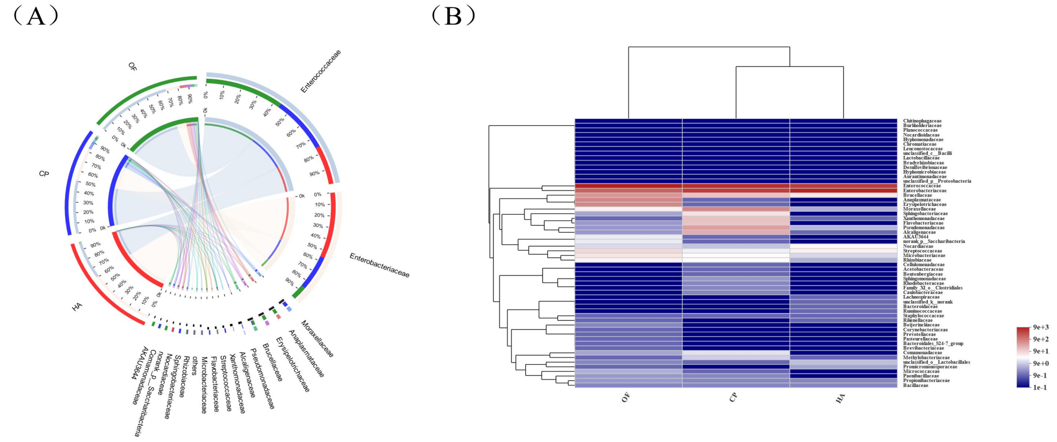

Figure S4. Circos (A) and distance heatmap (B) based on family level. Circos (A) shows distribution proportion of dominant species in each sample and distribution proportion of dominant species in different samples. The columns (B) represent the samples and the rows represent the bacterial assigned to the family level. Dendrograms of hierarchical cluster analysis grouping genera and samples are shown on the left and at the bottom, respectively. The color scale represents the normalized values of relative abundances by log10. HA, *H. armigera*; CP, *C. punctiferalis*; OF, *O. furnacalis*. Taxa with an abundance < 1% are included in 'Other'.

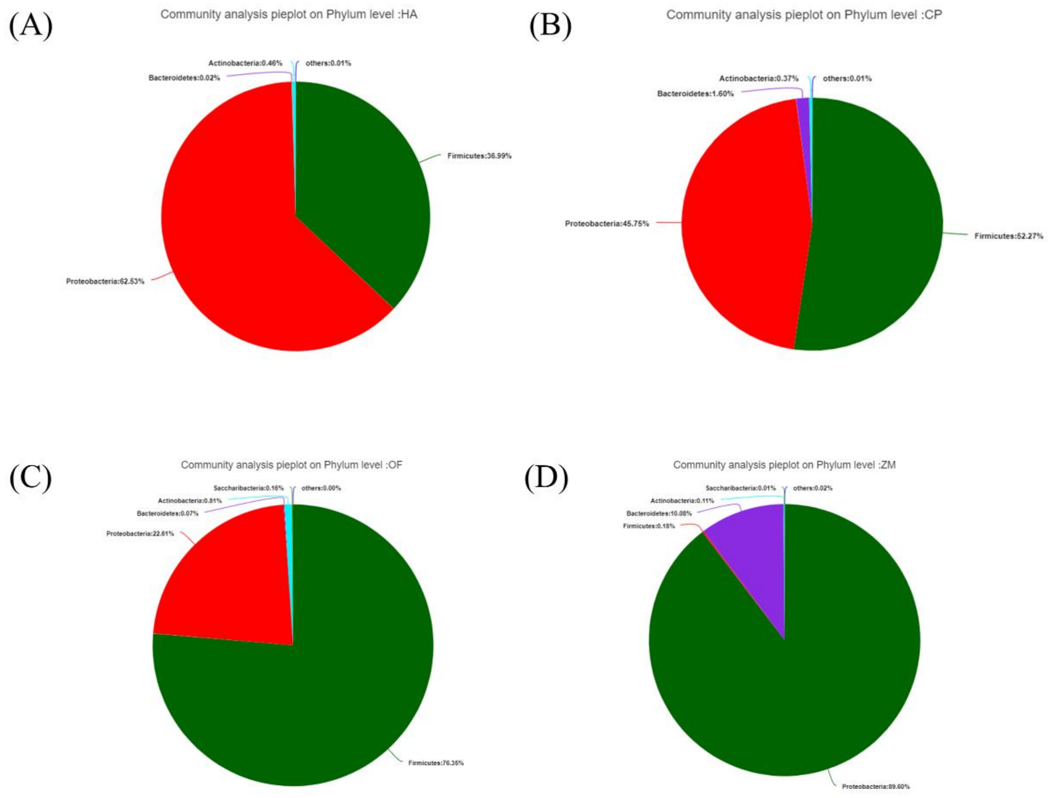

Figure S5. Pie chart of the intestinal and corn microbial community at phylum level. Different colors represent different phylum. HA, *H. armigera* (A); CP, *C. punctiferalis* (B); OF, *O. furnacalis* (C); ZM, *Z. mays* (D).

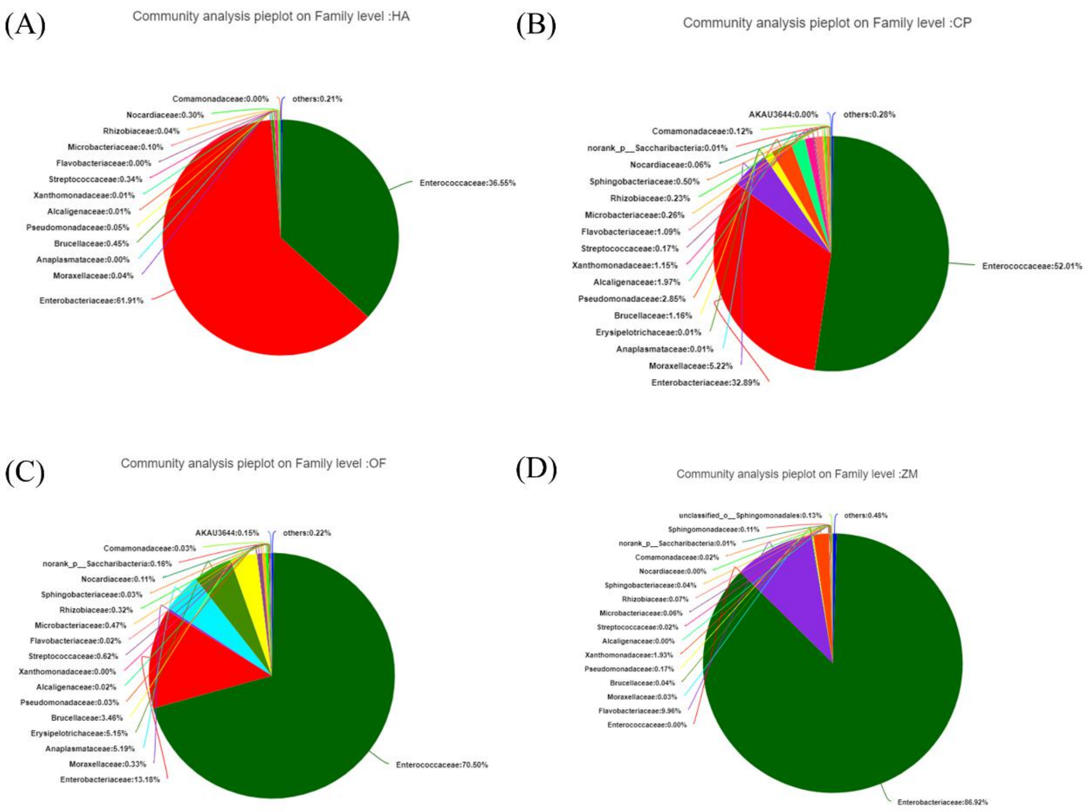

Figure S6. Pie chart of the intestinal and corn microbial community at family level. Different colors represent different families. HA, *H. armigera* (A); CP, *C. punctiferalis* (B); OF, *O. furnacalis* (C); ZM, *Z. mays* (D).

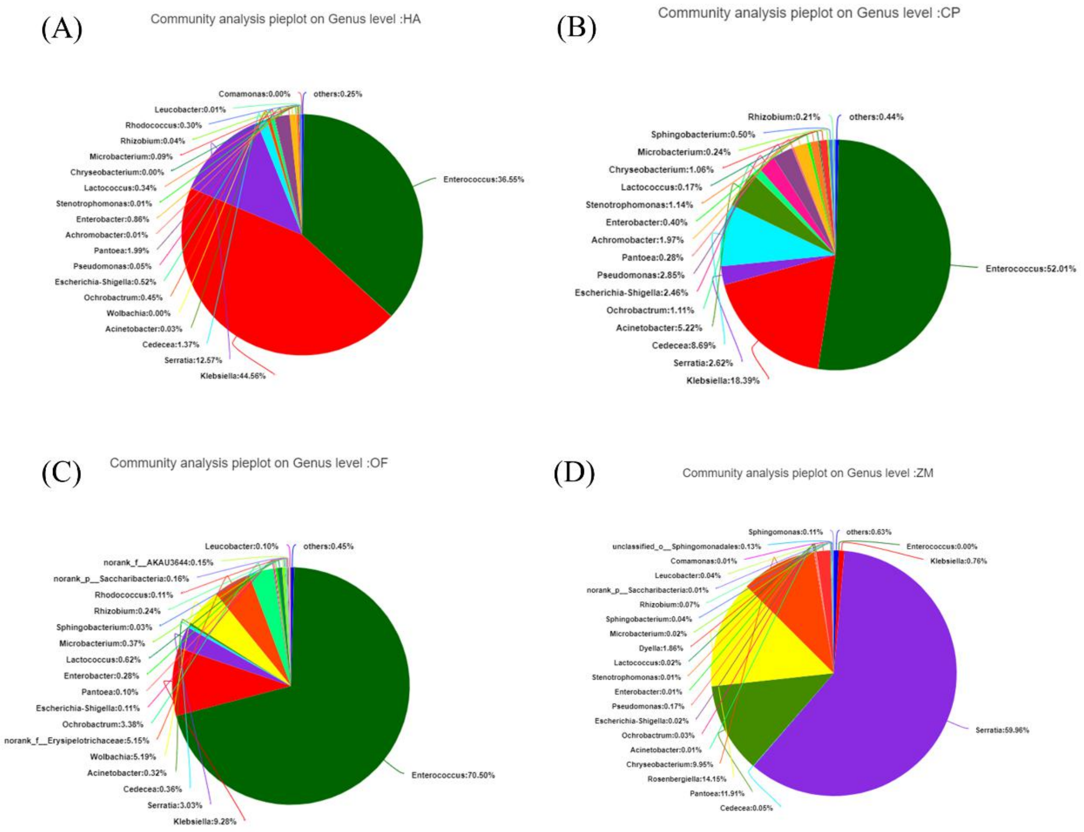

Figure S7. Pie chart of the intestinal and corn microbial community at genus level. Different colors represent different genus. HA, *H. armigera* (A); CP, *C. punctiferalis* (B); OF, *O. furnacalis* (C); ZM, *Z. mays* (D).

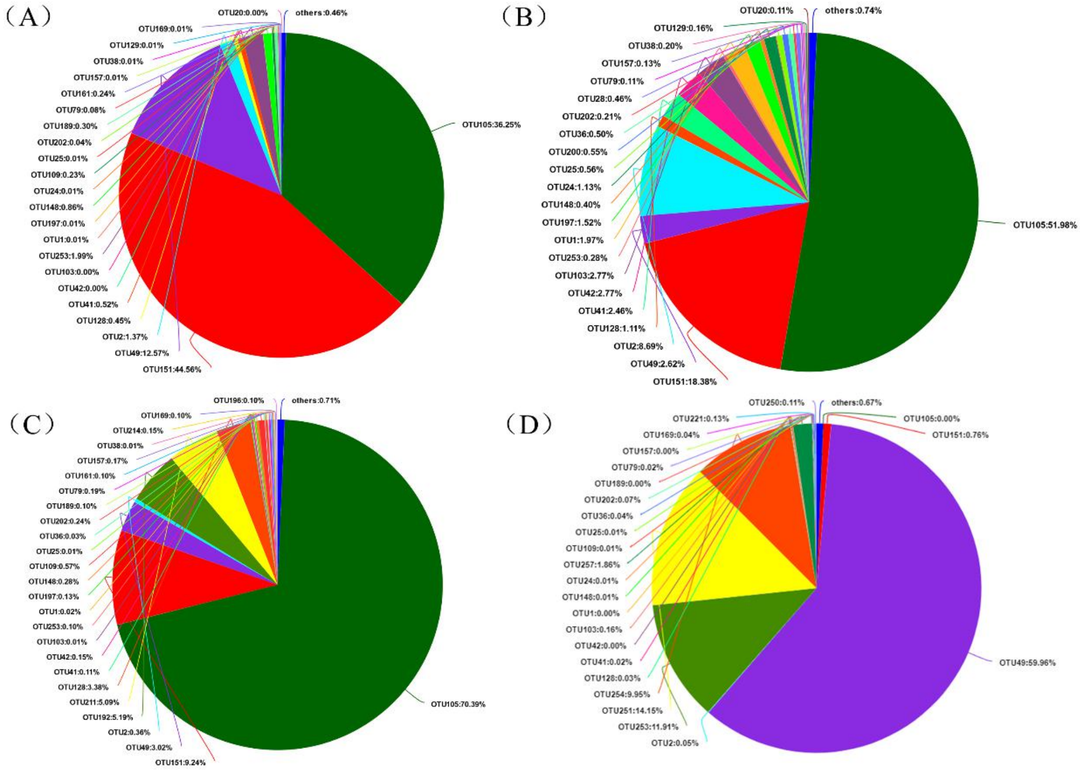

Figure S8. Pie chart of the intestinal and corn microbial community at OTU level. Different colors represent different OTUs. HA, *H. armigera* (A); CP, *C. punctiferalis* (B); OF, *O. furnacalis* (C); ZM, *Z. mays* (D).

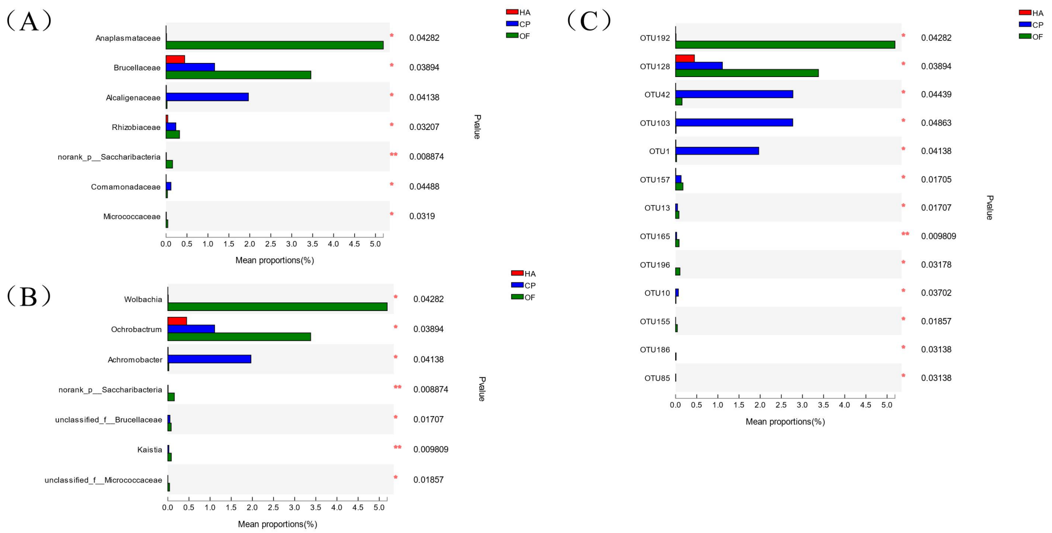

Figure S9. Significance difference test based on Kruskal-Wallis rank sum test method at family level (A), genus level (B) and OTU level (C). Y-axis represents the species name at a taxonomic level, X-axis represents the average relative abundance in different groups of species, and columns with different colors represent different groups. On the far right is the p value, * 0.01 < $P \leq 0.05$, ** 0.001 < $P \leq 0.01$. HA, *H. armigera*; CP, *C. punctiferalis*; OF, *O. furnacalis*.

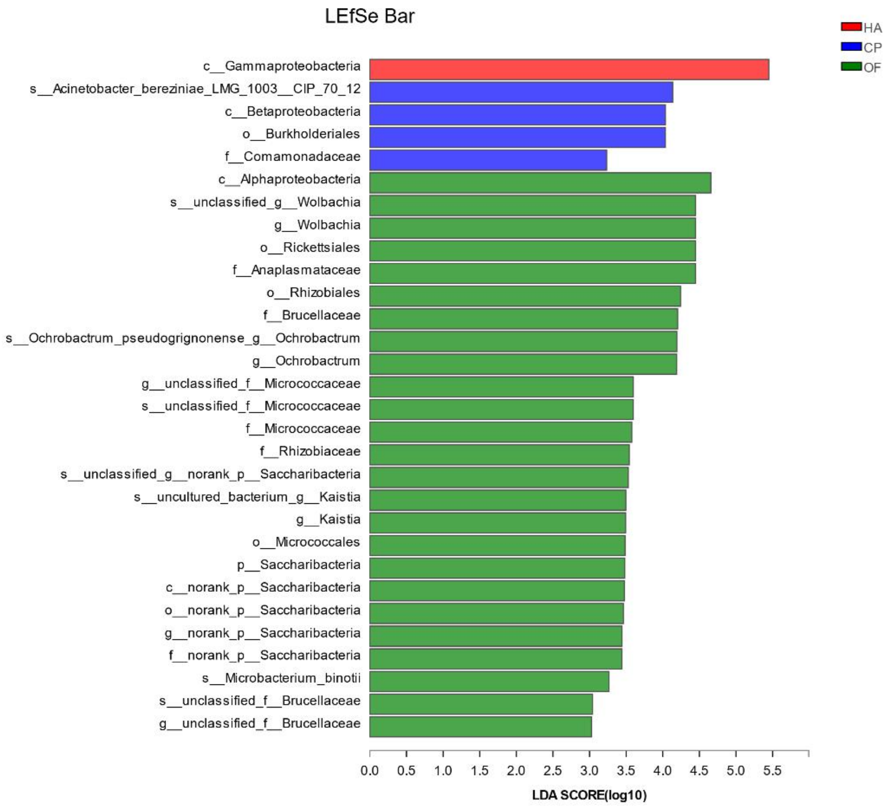

Figure S10. Linear discriminant analysis (LDA) of three pests. HA, *H. armigera*; CP, *C. punctiferalis*; OF, *O. furnacalis*.

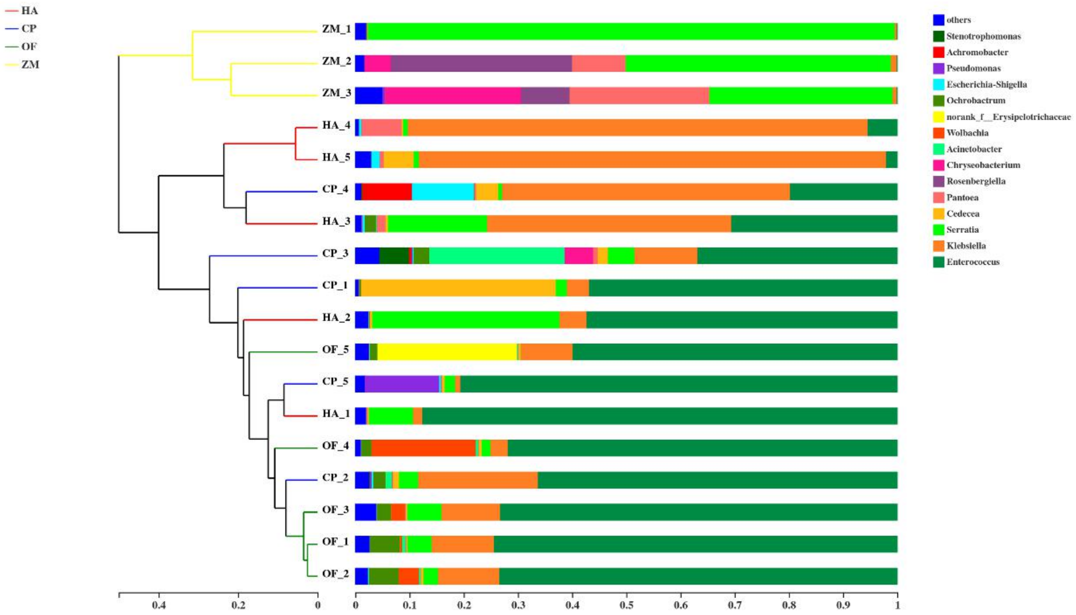

Figure S11. Clustering analysis based on Bray-Curtis distance algorithm at genus level. HA, *H. armigera*; CP, *C. punctiferalis*; OF, *O. furnacalis;* ZM, *Z. mays*.

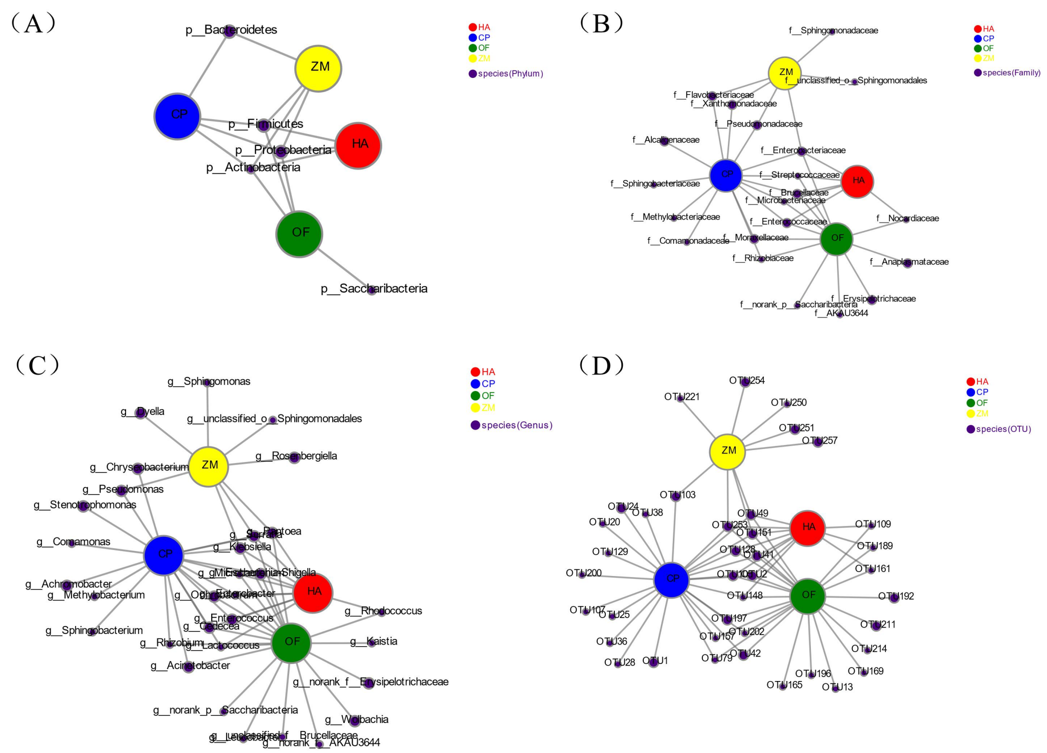

Figure S12. Co-occurrence network diagram of all samples at phylum level (A), family level (B), genus level (C) and OTU level (D). The network node represents a sample node or a species node, and the connection between the sample node and the species node represents that the sample contains the species. HA, *H. armigera*; CP, *C. punctiferalis*; OF, *O. furnacalis;* ZM, *Z. mays*.

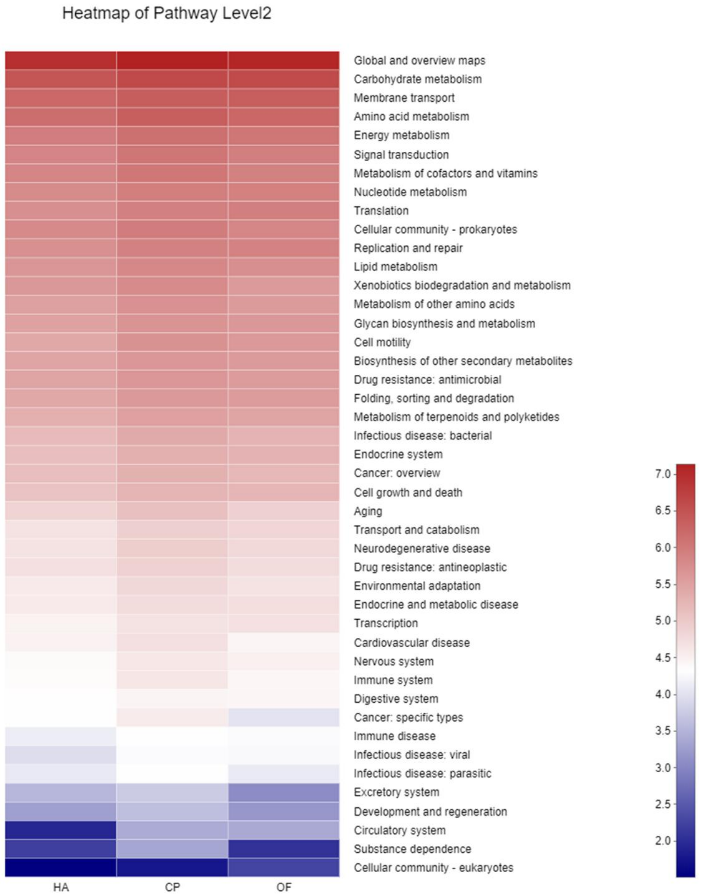

Figure S13. Pathway heatmap of Level2. Comparison of KEGG pathway function prediction of intestinal microbial community. HA, *H. armigera*; CP, *C. punctiferalis*; OF, *O. furnacalis*.

Table S1. Summary of multistage annotation information for three insect samples

Phylum	Class	Order	Family	Genus	OTU	CP (Total OTUs)	HA (Total OTUs)	OF (Total OTUs)	SUM
Firmicutes	Bacilli	Lactobacillales	Enterococcaceae	Enterococcus	OTU105	32121	22398	43496	98015
Proteobacteria	Gammaproteobacteria	Enterobacteriales	Enterobacteriaceae	Klebsiella	OTU151	11354	27533	5707	44594
Proteobacteria	Gammaproteobacteria	Enterobacteriales	Enterobacteriaceae	Serratia	OTU49	1617	7767	1869	11253
Proteobacteria	Gammaproteobacteria	Enterobacteriales	Enterobacteriaceae	Cedecea	OTU2	5372	845	220	6437
Proteobacteria	Alphaproteobacteria	Rickettsiales	Anaplasmataceae	Wolbachia	OTU192	7	1	3205	3213
Firmicutes	Erysipelotrichia	Erysipelotrichales	Erysipelotrichaceae	norank	OTU211	7	0	3148	3155
Proteobacteria	Alphaproteobacteria	Rhizobiales	Brucellaceae	Ochrobactrum	OTU128	684	275	2087	3046
Proteobacteria	Gammaproteobacteria	Enterobacteriales	Enterobacteriaceae	Escherichia	OTU41	1523	320	67	1910
Proteobacteria	Gammaproteobacteria	Pseudomonadales	Moraxellaceae	Acinetobacter	OTU42	1714	2	95	1811
Proteobacteria	Gammaproteobacteria	Pseudomonadales	Pseudomonadaceae	Pseudomonas	OTU103	1712	2	8	1722
Proteobacteria	Gammaproteobacteria	Enterobacteriales	Enterobacteriaceae	Pantoea	OTU253	175	1230	61	1466
Proteobacteria	Betaproteobacteria	Burkholderiales	Alcaligenaceae	Achromobacter	OTU1	1215	5	15	1235
Proteobacteria	Gammaproteobacteria	Pseudomonadales	Moraxellaceae	Acinetobacter	OTU197	938	5	83	1026
Proteobacteria	Gammaproteobacteria	Enterobacteriales	Enterobacteriaceae	Enterobacter	OTU148	248	534	173	955
Proteobacteria	Gammaproteobacteria	Xanthomonadales	Xanthomonadaceae	Stenotrophomonas	OTU24	699	5	3	707
Firmicutes	Bacilli	Lactobacillales	Streptococcaceae	Lactococcus	OTU109	36	143	352	531
Proteobacteria	Gammaproteobacteria	Pseudomonadales	Moraxellaceae	Acinetobacter	OTU25	347	5	9	361
Bacteroidetes	Flavobacteriia	Flavobacteriales	Flavobacteriaceae	Chryseobacterium	OTU200	342	0	6	348
Bacteroidetes	Sphingobacteriia	Sphingobacteriales	Sphingobacteriaceae	Sphingobacterium	OTU36	311	0	18	329
Proteobacteria	Alphaproteobacteria	Rhizobiales	Rhizobiaceae	Rhizobium	OTU202	129	26	146	301
Bacteroidetes	Flavobacteriia	Flavobacteriales	Flavobacteriaceae	Chryseobacterium	OTU28	286	1	1	288
Actinobacteria	Actinobacteria	Corynebacteriales	Nocardiaceae	Rhodococcus	OTU189	39	187	59	285

Actinobacteria	Actinobacteria	Micrococcales	Microbacteriaceae	Microbacterium	OTU79	65	49	119	233
Firmicutes	Bacilli	Lactobacillales	Enterococcaceae	Enterococcus	OTU161	1	151	62	214
Actinobacteria	Actinobacteria	Micrococcales	Microbacteriaceae	Microbacterium	OTU157	81	4	108	193
Proteobacteria	Gammaproteobacteria	Pseudomonadales	Moraxellaceae	Acinetobacter	OTU38	125	4	8	137
Proteobacteria	Gammaproteobacteria	Pseudomonadales	Moraxellaceae	Acinetobacter	OTU129	98	5	1	104
Actinobacteria	Actinobacteria	Micrococcales	AKAU3644	norank	OTU214	3	0	93	96
Actinobacteria	Actinobacteria	Micrococcales	Microbacteriaceae	Leucobacter	OTU169	15	7	62	84
Proteobacteria	Alphaproteobacteria	Rhizobiales	Brucellaceae	unclassified	OTU13	28	0	50	78
Proteobacteria	Betaproteobacteria	Burkholderiales	Comamonadaceae	Comamonas	OTU20	70	2	5	77
Firmicutes	Bacilli	Lactobacillales	Streptococcaceae	Lactococcus	OTU110	11	46	15	72
Proteobacteria	Alphaproteobacteria	Rhizobiales	Rhizobiaceae	Kaistia	OTU165	16	0	52	68
Saccharibacteria	norank	norank	norank	norank	OTU196	0	0	64	64
Firmicutes	Bacilli	Lactobacillales	Streptococcaceae	Lactococcus	OTU107	56	2	0	58
Proteobacteria	Alphaproteobacteria	Rhizobiales	Methylobacteriaceae	Methylobacterium	OTU106	48	0	0	48
Proteobacteria	Gammaproteobacteria	Pseudomonadales	Pseudomonadaceae	Pseudomonas	OTU10	41	0	6	47
Firmicutes	Erysipelotrichia	Erysipelotrichales	Erysipelotrichaceae	norank	OTU204	0	0	37	37
Firmicutes	Bacilli	Lactobacillales	unclassified	unclassified	OTU153	2	24	10	36
Proteobacteria	Gammaproteobacteria	Enterobacteriales	Enterobacteriaceae	Klebsiella	OTU14	7	3	26	36
Firmicutes	Bacilli	Lactobacillales	Streptococcaceae	Lactococcus	OTU184	0	18	17	35
Firmicutes	Bacilli	Lactobacillales	Enterococcaceae	Enterococcus	OTU130	12	15	4	31
Actinobacteria	Actinobacteria	Propionibacteriales	Propionibacteriaceae	Propionibacterium	OTU216	9	10	12	31
Proteobacteria	Gammaproteobacteria	Enterobacteriales	Enterobacteriaceae	unclassified	OTU16	10	15	4	29
Actinobacteria	Actinobacteria	Micrococcales	Promicromonosporaceae	Cellulosimicrobium	OTU132	0	22	5	27
Actinobacteria	Actinobacteria	Micrococcales	Micrococcaceae	unclassified	OTU155	1	0	26	27
Bacteroidetes	Flavobacteriia	Flavobacteriales	Flavobacteriaceae	Chryseobacterium	OTU29	26	0	0	26
Firmicutes	Bacilli	Lactobacillales	Enterococcaceae	Enterococcus	OTU108	4	18	1	23

Firmicutes	Bacilli	Bacillales	Bacillaceae	Anoxybacillus	OTU125	7	8	8	23
Proteobacteria	Gammaproteobacteria	Pseudomonadales	Pseudomonadaceae	Pseudomonas	OTU133	0	21	2	23
Proteobacteria	Alphaproteobacteria	Sphingomonadales	Sphingomonadaceae	Novosphingobium	OTU32	17	0	1	18
Bacteroidetes	Flavobacteriia	Flavobacteriales	Flavobacteriaceae	Empedobacter	OTU31	18	0	0	18
Firmicutes	Bacilli	Bacillales	Paenibacillaceae	Paenibacillus	OTU212	3	0	14	17
Saccharibacteria	norank	norank	norank	norank	OTU199	1	0	16	17
Saccharibacteria	norank	norank	norank	norank	OTU12	2	0	12	14
Proteobacteria	Alphaproteobacteria	Rhizobiales	Methylobacteriaceae	Methylobacterium	OTU210	4	6	4	14
Firmicutes	Bacilli	Lactobacillales	unclassified	unclassified	OTU9	2	11	1	14
Firmicutes	Bacilli	Bacillales	Staphylococcaceae	Staphylococcus	OTU246	6	3	3	12
Proteobacteria	Gammaproteobacteria	Enterobacteriales	Enterobacteriaceae	Rosenbergiella	OTU251	8	2	2	12
Proteobacteria	Gammaproteobacteria	Enterobacteriales	Enterobacteriaceae	Pectobacterium	OTU175	3	3	5	11
Proteobacteria	Gammaproteobacteria	Enterobacteriales	Enterobacteriaceae	unclassified	OTU178	3	0	7	10
Proteobacteria	Alphaproteobacteria	Caulobacterales	Caulobacteraceae	Caulobacter	OTU102	9	1	0	10
Proteobacteria	Alphaproteobacteria	Rhodobacterales	Rhodobacteraceae	unclassified	OTU15	8	0	0	8
Proteobacteria	Gammaproteobacteria	Xanthomonadales	Xanthomonadaceae	Stenotrophomonas	OTU40	8	0	0	8
Actinobacteria	Actinobacteria	Corynebacteriales	Nocardiaceae	Rhodococcus	OTU186	0	0	8	8
Bacteroidetes	Flavobacteriia	Flavobacteriales	Flavobacteriaceae	Empedobacter	OTU174	0	0	7	7
Proteobacteria	Alphaproteobacteria	Rhizobiales	Beijerinckiaceae	Qingshengfania	OTU173	0	0	7	7
Proteobacteria	Gammaproteobacteria	Pseudomonadales	Pseudomonadaceae	Pseudomonas	OTU183	5	1	1	7
Firmicutes	Bacilli	Bacillales	Paenibacillaceae	Ammoniphilus	OTU194	2	2	3	7
Proteobacteria	Betaproteobacteria	Burkholderiales	Comamonadaceae	Comamonas	OTU179	0	0	6	6
Bacteroidetes	Bacteroidia	Bacteroidales	Rikenellaceae	Rikenellaceae	OTU172	1	2	3	6
Proteobacteria	Gammaproteobacteria	Pseudomonadales	Pseudomonadaceae	Pseudomonas	OTU111	0	6	0	6
unclassified	unclassified	unclassified	unclassified	unclassified	OTU150	0	5	0	5
Proteobacteria	Gammaproteobacteria	Pasteurellales	Pasteurellaceae	Haemophilus	OTU116	0	2	3	5

Proteobacteria	Betaproteobacteria	Burkholderiales	Comamonadaceae	Lampropedia	OTU168	2	0	3	5
Proteobacteria	Gammaproteobacteria	Pseudomonadales	Moraxellaceae	Acinetobacter	OTU195	1	0	3	4
Actinobacteria	Actinobacteria	Corynebacteriales	Corynebacteriaceae	Corynebacterium	OTU248	0	2	2	4
Firmicutes	Clostridia	Clostridiales	Family_XI_Clostridiales	Ezakiella	OTU33	4	0	0	4
Actinobacteria	Actinobacteria	Micrococcales	Cellulomonadaceae	Cellulomonas	OTU223	3	0	1	4
Proteobacteria	Alphaproteobacteria	Rhodospirillales	Acetobacteraceae	Roseomonas	OTU27	2	0	2	4
Proteobacteria	Gammaproteobacteria	Pseudomonadales	Moraxellaceae	Acinetobacter	OTU19	4	0	0	4
Proteobacteria	Gammaproteobacteria	Pseudomonadales	Moraxellaceae	Enhydrobacter	OTU242	0	2	2	4
Firmicutes	Bacilli	unclassified	unclassified	unclassified	OTU209	2	0	2	4
Firmicutes	Bacilli	Lactobacillales	Leuconostocaceae	Leuconostoc	OTU122	2	2	0	4
Firmicutes	Bacilli	Bacillales	Bacillaceae	Bacillus	OTU198	3	1	0	4
Proteobacteria	Alphaproteobacteria	Rhizobiales	Brucellaceae	unclassified	OTU17	4	0	0	4
Actinobacteria	Actinobacteria	Micrococcales	Beutenbergiaceae	Salana	OTU34	4	0	0	4
Firmicutes	Bacilli	Bacillales	Paenibacillaceae	Paenibacillus	OTU85	4	0	0	4
Saccharibacteria	norank	norank	norank	norank	OTU164	1	0	3	4
Proteobacteria	Betaproteobacteria	Burkholderiales	Comamonadaceae	Comamonas	OTU238	1	0	3	4
Actinobacteria	Actinobacteria	Micrococcales	Micrococcaceae	Arthrobacter	OTU104	4	0	0	4
Firmicutes	Clostridia	Clostridiales	Lachnospiraceae	norank	OTU113	0	3	0	3
Firmicutes	Clostridia	Clostridiales	Family_XI_Clostridiales	Peptoniphilus	OTU35	3	0	0	3
Proteobacteria	Betaproteobacteria	Burkholderiales	Comamonadaceae	Delftia	OTU185	0	0	3	3
Actinobacteria	Actinobacteria	Micrococcales	Microbacteriaceae	Leucobacter	OTU127	2	1	0	3
Proteobacteria	Alphaproteobacteria	Rhizobiales	Hyphomicrobiaceae	Devosia	OTU167	1	0	2	3
Firmicutes	Clostridia	Clostridiales	Ruminococcaceae	Ruminococcaceae	OTU120	0	3	0	3
Firmicutes	Bacilli	Bacillales	Bacillaceae	Bacillus	OTU48	1	2	0	3
Actinobacteria	Actinobacteria	Corynebacteriales	Corynebacteriaceae	Lawsonella	OTU217	1	0	2	3
Proteobacteria	Alphaproteobacteria	Rhizobiales	Beijerinckiaceae	Camelimonas	OTU208	1	0	2	3

Proteobacteria	unclassified	unclassified	unclassified	unclassified	OTU188	0	1	2	3
Firmicutes	Bacilli	Lactobacillales	Streptococcaceae	Streptococcus	OTU227	0	1	2	3
Proteobacteria	Alphaproteobacteria	Rhizobiales	Bradyrhizobiaceae	Bosea	OTU180	1	0	2	3
Actinobacteria	Actinobacteria	Micrococcales	Brevibacteriaceae	Brevibacterium	OTU193	0	0	3	3
unclassified	unclassified	unclassified	unclassified	unclassified	OTU158	0	0	2	2
Bacteroidetes	Bacteroidia	Bacteroidales	Bacteroidales	norank	OTU3	1	0	1	2
Bacteroidetes	Bacteroidia	Bacteroidales	Prevotellaceae	Prevotellaceae	OTU177	0	0	2	2
Bacteroidetes	Bacteroidia	Bacteroidales	Rikenellaceae	Rikenella	OTU166	0	2	0	2
Bacteroidetes	Bacteroidia	Bacteroidales	Bacteroidaceae	Bacteroides	OTU7	0	2	0	2
Bacteroidetes	Bacteroidia	Bacteroidales	Prevotellaceae	Alloprevotella	OTU135	1	0	1	2
Proteobacteria	Gammaproteobacteria	Pseudomonadales	Pseudomonadaceae	Pseudomonas	OTU90	2	0	0	2
Firmicutes	Clostridia	Clostridiales	Ruminococcaceae	Anaerotruncus	OTU126	0	2	0	2
Proteobacteria	Gammaproteobacteria	Xanthomonadales	Xanthomonadaceae	Dyella	OTU257	2	0	0	2
Saccharibacteria	norank	norank	norank	norank	OTU213	1	0	1	2
Proteobacteria	Alphaproteobacteria	Rhizobiales	Aurantimonadaceae	Aureimonas	OTU170	1	1	0	2
Proteobacteria	Alphaproteobacteria	Rhizobiales	Bradyrhizobiaceae	Bradyrhizobium	OTU114	0	2	0	2
Firmicutes	Clostridia	Clostridiales	Family_XI_Clostridiales	Anaerococcus	OTU234	2	0	0	2
Bacteroidetes	Bacteroidia	Bacteroidales	Bacteroidaceae	Bacteroides	OTU119	0	2	0	2
Bacteroidetes	Bacteroidia	Bacteroidales	Bacteroidales	norank	OTU145	0	0	2	2
Firmicutes	Clostridia	Clostridiales	Lachnospiraceae	Lachnospiraceae	OTU159	1	0	1	2
Firmicutes	Bacilli	Bacillales	Planococcaceae	Solibacillus	OTU233	2	0	0	2
Bacteroidetes	Bacteroidia	Bacteroidales	Bacteroidales	norank	OTU154	0	0	2	2
Bacteroidetes	Flavobacteriia	Flavobacteriales	Flavobacteriaceae	Chryseobacterium	OTU254	2	0	0	2
Bacteroidetes	Bacteroidia	Bacteroidales	Bacteroidales	norank	OTU176	1	0	0	1
Proteobacteria	Betaproteobacteria	Burkholderiales	Comamonadaceae	Aquabacterium	OTU201	0	0	1	1
Bacteroidetes	Flavobacteriia	Flavobacteriales	Flavobacteriaceae	Elizabethkingia	OTU68	1	0	0	1

Bacteroidetes	Bacteroidia	Bacteroidales	Bacteroidales	norank	OTU140	0	0	1	1
Firmicutes	Bacilli	Bacillales	Paenibacillaceae	Paenibacillus	OTU11	1	0	0	1
Proteobacteria	Alphaproteobacteria	Caulobacterales	Caulobacteraceae	Brevundimonas	OTU205	1	0	0	1
Proteobacteria	Alphaproteobacteria	Sphingomonadales	Sphingomonadaceae	Sphingobium	OTU121	0	1	0	1
Proteobacteria	Alphaproteobacteria	Caulobacterales	Hyphomonadaceae	norank	OTU112	0	1	0	1
Proteobacteria	Alphaproteobacteria	Rhizobiales	Rhizobiaceae	Rhizobium	OTU187	0	0	1	1
Actinobacteria	Actinobacteria	Corynebacteriales	Corynebacteriaceae	Corynebacterium	OTU181	0	0	1	1
Bacteroidetes	Bacteroidia	Bacteroidales	Bacteroidales	norank	OTU171	0	0	1	1
Proteobacteria	Betaproteobacteria	Burkholderiales	Burkholderiaceae	Burkholderia	OTU75	1	0	0	1
Actinobacteria	Actinobacteria	Propionibacteriales	Nocardioidaceae	Nocardioides	OTU182	1	0	0	1
Proteobacteria	Gammaproteobacteria	Enterobacteriales	Enterobacteriaceae	Serratia	OTU160	0	0	1	1
Actinobacteria	Actinobacteria	Propionibacteriales	Propionibacteriaceae	Propionibacterium	OTU5	0	0	1	1
Actinobacteria	Actinobacteria	Corynebacteriales	Corynebacteriaceae	Corynebacterium	OTU52	0	0	1	1
Bacteroidetes	Bacteroidia	Bacteroidales	Bacteroidales	norank	OTU26	0	1	0	1
Proteobacteria	Alphaproteobacteria	Rhodobacterales	Rhodobacteraceae	unclassified	OTU144	0	1	0	1
Proteobacteria	Gammaproteobacteria	Chromatiales	Chromatiaceae	Rheinheimera	OTU146	1	0	0	1
Bacteroidetes	Sphingobacteriia	Sphingobacteriales	Chitinophagaceae	Taibaiella	OTU229	1	0	0	1
Proteobacteria	Deltaproteobacteria	Desulfovibrionales	Desulfovibrionaceae	Desulfovibrio	OTU6	1	0	0	1
Proteobacteria	Gammaproteobacteria	Enterobacteriales	Enterobacteriaceae	Serratia	OTU8	0	1	0	1
Proteobacteria	Betaproteobacteria	Burkholderiales	Burkholderiaceae	Pandoraea	OTU207	0	0	1	1
Firmicutes	Clostridia	Clostridiales	Lachnospiraceae	unclassified	OTU124	0	1	0	1
Firmicutes	Bacilli	Lactobacillales	Lactobacillaceae	Lactobacillus	OTU249	0	1	0	1
Bacteroidetes	Bacteroidia	Bacteroidales	Bacteroidales	norank	OTU123	0	1	0	1
Proteobacteria	Alphaproteobacteria	Caulobacterales	Caulobacteraceae	Brevundimonas	OTU37	0	0	1	1
Firmicutes	Clostridia	Clostridiales	Family_XI_Clostridiales	Anaerococcus	OTU118	1	0	0	1
Firmicutes	Clostridia	Clostridiales	Family_XI_Clostridiales	Finegoldia	OTU131	1	0	0	1

Proteobacteria	Alphaproteobacteria	Rhodospirillales	Acetobacteraceae	Asaia	OTU98	1	0	0	1
Proteobacteria	Alphaproteobacteria	Rhizobiales	Methylobacteriaceae	Methylobacterium	OTU100	0	0	1	1
Firmicutes	Clostridia	Clostridiales	Ruminococcaceae	unclassified	OTU117	0	1	0	1

The first five columns were the species names OF the corresponding phylum, class, order, family, and genus, and the sixth column was the OTU numbering information corresponding to this species. CP (total OTUs) was the abundance sum (sequence number) of each OTU corresponding to all samples of *C. punctiferalis*, HA (total OTUs) was the abundance sum (sequence number) of each OTU corresponding to all samples of *H. armigera*, OF (total OTUs) was the abundance sum (sequence number) of each OTU corresponding to all samples of *O. furnacalis*, and SUM was the abundance sum of this species in all samples of three insects.

Table S2. Summary of Multi-level Annotation Information of Corn Samples

Phylum	Class	Order	Family	Genus	OTU	ZM(total OTUs)
Proteobacteria	Gammaproteobacteria	Enterobacteriales	Enterobacteriaceae	Serratia	OTU49	22230
Proteobacteria	Gammaproteobacteria	Enterobacteriales	Enterobacteriaceae	Rosenbergiella	OTU251	5247
Proteobacteria	Gammaproteobacteria	Enterobacteriales	Enterobacteriaceae	Pantoea	OTU253	4414
Bacteroidetes	Flavobacteriia	Flavobacteriales	Flavobacteriaceae	Chryseobacterium	OTU254	3689
Proteobacteria	Gammaproteobacteria	Xanthomonadales	Xanthomonadaceae	Dyella	OTU257	690
Proteobacteria	Gammaproteobacteria	Enterobacteriales	Enterobacteriaceae	Klebsiella	OTU151	282
Proteobacteria	Gammaproteobacteria	Pseudomonadales	Pseudomonadaceae	Pseudomonas	OTU103	60
Proteobacteria	Alphaproteobacteria	Sphingomonadales	unclassified	unclassified	OTU221	50
Proteobacteria	Alphaproteobacteria	Sphingomonadales	Sphingomonadaceae	Sphingomonas	OTU250	41
Proteobacteria	Alphaproteobacteria	Rhizobiales	Rhizobiaceae	Rhizobium	OTU202	27
Bacteroidetes	Cytophagia	Cytophagales	Cytophagaceae	Sporocytophaga	OTU232	26
Proteobacteria	Gammaproteobacteria	Enterobacteriales	Enterobacteriaceae	unclassified	OTU16	20
Proteobacteria	Betaproteobacteria	Burkholderiales	Oxalobacteraceae	norank	OTU239	18
Proteobacteria	Gammaproteobacteria	Enterobacteriales	Enterobacteriaceae	Cedecea	OTU2	17
Bacteroidetes	Sphingobacteriia	Sphingobacteriales	Sphingobacteriaceae	Sphingobacterium	OTU36	14
Actinobacteria	Actinobacteria	Micrococcales	Microbacteriaceae	Leucobacter	OTU169	13
Proteobacteria	Alphaproteobacteria	Rhizobiales	Brucellaceae	Ochrobactrum	OTU128	12
Proteobacteria	Gammaproteobacteria	Xanthomonadales	Xanthomonadaceae	Xanthomonas	OTU255	12
Firmicutes	Bacilli	Lactobacillales	Lactobacillaceae	Lactobacillus	OTU231	11
Actinobacteria	Actinobacteria	Propionibacteriales	Propionibacteriaceae	Propionibacterium	OTU216	10
Proteobacteria	Alphaproteobacteria	Rhodobacterales	Rhodobacteraceae	Rubellimicrobium	OTU241	9
Proteobacteria	Alphaproteobacteria	Rhizobiales	Methylobacteriaceae	Methylobacterium	OTU210	9
Chloroflexi	Anaerolineae	Anaerolineales	Anaerolineaceae	norank	OTU237	9
Firmicutes	Bacilli	Bacillales	Planococcaceae	Solibacillus	OTU233	9

Proteobacteria	Alphaproteobacteria	Rhodospirillales	Acetobacteraceae	Asaia	OTU98	9
Proteobacteria	Gammaproteobacteria	Enterobacteriales	Enterobacteriaceae	Escherichia-Shigella	OTU41	8
Actinobacteria	Actinobacteria	Micrococcales	Microbacteriaceae	Microbacterium	OTU79	8
Proteobacteria	Gammaproteobacteria	Xanthomonadales	Xanthomonadaceae	Pseudoxanthomonas	OTU256	7
Firmicutes	Bacilli	Bacillales	Bacillaceae	Bacillus	OTU4	7
Firmicutes	Clostridia	Clostridiales	Family_XI_Clostridiales	Peptoniphilus	OTU235	7
Proteobacteria	Betaproteobacteria	Burkholderiales	Comamonadaceae	Aquabacterium	OTU201	6
Firmicutes	Bacilli	Bacillales	Bacillaceae	Bacillus	OTU198	6
Firmicutes	Bacilli	Lactobacillales	Streptococcaceae	Lactococcus	OTU109	5
Firmicutes	Bacilli	Bacillales	Staphylococcaceae	Staphylococcus	OTU246	5
Proteobacteria	Gammaproteobacteria	Xanthomonadales	Xanthomonadaceae	Stenotrophomonas	OTU24	5
Proteobacteria	Gammaproteobacteria	Enterobacteriales	Enterobacteriaceae	Enterobacter	OTU148	5
Proteobacteria	Alphaproteobacteria	Caulobacterales	Caulobacteraceae	Caulobacter	OTU102	5
Proteobacteria	Alphaproteobacteria	Rhodobacterales	Rhodobacteraceae	unclassified	OTU15	4
Firmicutes	Bacilli	Lactobacillales	Streptococcaceae	Lactococcus	OTU107	4
Bacteroidetes	Flavobacteriia	Flavobacteriales	Flavobacteriaceae	Flavobacterium	OTU240	4
Proteobacteria	Gammaproteobacteria	Pseudomonadales	Moraxellaceae	Enhydrobacter	OTU242	4
Proteobacteria	Gammaproteobacteria	Pseudomonadales	Moraxellaceae	Acinetobacter	OTU25	4
Bacteroidetes	Cytophagia	Cytophagales	unclassified	unclassified	OTU222	4
Proteobacteria	Betaproteobacteria	Burkholderiales	Oxalobacteraceae	Massilia	OTU23	3
Firmicutes	Clostridia	Clostridiales	Family_XI_Clostridiales	Ezakiella	OTU33	3
Saccharibacteria	norank	norank	norank	norank	OTU12	3
Firmicutes	Clostridia	Clostridiales	Family_XI_Clostridiales	Anaerococcus	OTU234	3
Proteobacteria	Betaproteobacteria	Rhodocyclales	Rhodocyclaceae	Zoogloea	OTU228	3
Proteobacteria	Betaproteobacteria	Burkholderiales	Comamonadaceae	Comamonas	OTU238	3
Actinobacteria	Actinobacteria	Corynebacteriales	Corynebacteriaceae	Corynebacterium	OTU248	2

Proteobacteria	Alphaproteobacteria	Rhizobiales	Rhizobiales_Incertae_Sedis	Rhizomicrobium	OTU230	2
Proteobacteria	Gammaproteobacteria	Pseudomonadales	Pseudomonadaceae	Pseudomonas	OTU88	2
Proteobacteria	Alphaproteobacteria	Rhizobiales	Phyllobacteriaceae	Aquamicrobium	OTU224	2
Firmicutes	Clostridia	Clostridiales	Family_XI_Clostridiales	Finegoldia	OTU131	2
Proteobacteria	Alphaproteobacteria	Rhizobiales	Methylobacteriaceae	Methylobacterium	OTU100	2
Actinobacteria	Actinobacteria	Streptomycetales	Streptomycetaceae	Streptomyces	OTU89	2
Firmicutes	Bacilli	Lactobacillales	Enterococcaceae	Enterococcus	OTU105	1
Proteobacteria	Betaproteobacteria	Burkholderiales	Alcaligenaceae	Achromobacter	OTU1	1
Actinobacteria	Actinobacteria	Micrococcales	Microbacteriaceae	Microbacterium	OTU157	1
Proteobacteria	Gammaproteobacteria	Pseudomonadales	Moraxellaceae	Perlucidibaca	OTU156	1
Firmicutes	Bacilli	Bacillales	Bacillaceae	Oceanobacillus	OTU65	1
Proteobacteria	Alphaproteobacteria	Rhizobiales	Hyphomicrobiaceae	Devosia	OTU167	1
Firmicutes	Bacilli	Bacillales	Bacillaceae	Bacillus	OTU48	1
Firmicutes	Bacilli	Lactobacillales	Leuconostocaceae	Leuconostoc	OTU122	1
Proteobacteria	Gammaproteobacteria	Pseudomonadales	Pseudomonadaceae	Pseudomonas	OTU183	1
Proteobacteria	Alphaproteobacteria	Rhizobiales	Brucellaceae	unclassified	OTU13	1
Proteobacteria	Gammaproteobacteria	Enterobacteriales	Enterobacteriaceae	Serratia	OTU8	1
Actinobacteria	Actinobacteria	Micrococcales	Beutenbergiaceae	Salana	OTU34	1
Actinobacteria	Actinobacteria	Corynebacteriales	Nocardiaceae	Rhodococcus	OTU189	1
Firmicutes	Bacilli	Lactobacillales	Lactobacillaceae	Lactobacillus	OTU249	1
Actinobacteria	Actinobacteria	Micrococcales	Micrococcaceae	Arthrobacter	OTU104	1
Proteobacteria	Gammaproteobacteria	Pseudomonadales	Moraxellaceae	Acinetobacter	OTU42	1

The first five columns were the species names of the corresponding phylum, class, order, family and genus, and the sixth column was the OTU numbering information corresponding to that species. ZM(total OTUs) was the abundance sum (sequence number) of each OTU corresponding to all samples of maize.
